# Metal-coupled folding as the driving force for the extreme stability of Rad50 zinc hook dimer assembly

**DOI:** 10.1038/srep36346

**Published:** 2016-11-03

**Authors:** Tomasz Kochańczyk, Michał Nowakowski, Dominika Wojewska, Anna Kocyła, Andrzej Ejchart, Wiktor Koźmiński, Artur Krężel

**Affiliations:** 1 Department of Chemical Biology, Faculty of Biotechnology, University of Wrocław, Joliot-Curie 14a, 50-383 Wrocław, Poland; 2 Biological and Chemical Research Center, Faculty of Chemistry, University of Warsaw, Żwirki i Wigury 101, 02-089 Warsaw, Poland; 3 Institute of Biochemistry and Biophysics, Polish Academy of Sciences, Pawińskiego 5a, 02-106, Warsaw, Poland

## Abstract

The binding of metal ions at the interface of protein complexes presents a unique and poorly understood mechanism of molecular assembly. A remarkable example is the Rad50 zinc hook domain, which is highly conserved and facilitates the Zn^2+^-mediated homodimerization of Rad50 proteins. Here, we present a detailed analysis of the structural and thermodynamic effects governing the formation and stability (log*K*_12_ = 20.74) of this evolutionarily conserved protein assembly. We have dissected the determinants of the stability contributed by the small β-hairpin of the domain surrounding the zinc binding motif and the coiled-coiled regions using peptides of various lengths from 4 to 45 amino acid residues, alanine substitutions and peptide bond-to-ester perturbations. In the studied series of peptides, an >650 000-fold increase of the formation constant of the dimeric complex arises from favorable enthalpy because of the increased acidity of the cysteine thiols in metal-free form and the structural properties of the dimer. The dependence of the enthalpy on the domain fragment length is partially compensated by the entropic penalty of domain folding, indicating enthalpy-entropy compensation. This study facilitates understanding of the metal-mediated protein-protein interactions in which the metal ion is critical for the tight association of protein subunits.

Transition metal ions play an important role in facilitating the diverse functions of proteins. Among all transition metal ions, zinc (formally Zn^2+^) is the most widespread cofactor in proteins^1^,^2^. Based on their function, the zinc binding domains in proteins have been categorized into catalytic, structural and regulatory classes. Another classification scheme is based on the architecture of particular zinc binding domains, namely, the number of polypeptide chains or protein subunits that contribute to Zn^2+^ binding. The vast majority of known zinc domains, such as the zinc fingers and catalytic domains in enzymes, have an intramolecular binding architecture, in which all protein-derived zinc ligands are located within a single polypeptide chain^1^,^2^. Alternatively, Zn^2+^ can be intermolecularly bound, i.e., by two or more peptide chains, bridging these molecules to form a protein assembly^3^,^4^,^5^, as in the case of the Rad50 dimer protein^6^ or a higher-order assembly^7^,^8^.

Rad50 protein is a member of an evolutionarily conserved Mre11 complex (Mre11, Rad50, and Nbs1 in eukaryotes) that plays a pivotal role in the DNA damage response, including cell cycle checkpoint activation in response to DNA double-strand breaks (DSBs), DSB repair and telomere maintenance^9^. Rad50 is a rod-shaped molecule with a long antiparallel coiled-coil protruding from the globular ATPase/DNA-binding domain. The apex of the coiled-coil contains a small loop with a conserved Cys-Xaa-Xaa-Cys (CXXC) motif. Rad50 forms a homodimer through two pairs of cysteines from CXXC motifs that contribute to form an intermolecular zinc complex known as zinc hook domain (Fig. 1a). The zinc hook is conserved in Rad50 homologs in all forms of life, from Archaea to humans and even viruses^10^. The global influence of the zinc hook on the functions of Rad50 is universal as disruption of the Zn^2+^ binding to the zinc hook is lethal^11^,^12^. The destabilization of the zinc hook complex has a long-range allosteric effect on the several hundred angstroms distant globular domain of Rad50, affecting the DNA damage response, DNA recombination, telomere integrity and meiosis. The precise role of the zinc hook complex in these processes and how the stability of this complex relates to its function are unknown^11^,^13^.

Several intramolecular Zn^2+^ binding domains, such as zinc fingers, have been studied in considerable detail regarding metal-binding affinity, thermodynamics and structure-stability relationships^14^,^15^. In contrast, there is a lack of analyses aimed at understanding the energetic and structural effects accompanying the formation of protein-interface Zn^2+^ binding domains, although this aspect is crucial for understanding the roles of such domains in biological processes^16^.

Specific protein-protein interactions are typically stabilized through an extensive network of non-covalent interactions that outcompete the energetic cost attributed to the loss of entropy and the dehydration of protein surfaces^17^,^18^. The binding of metal ions at the protein-protein interface significantly contributes to the complex formation thermodynamics, and may promote molecular recognition and induce changes in the conformation of the resulting complex. To achieve sufficient stability and specificity, the protein-protein interface containing only non-covalent interactions must bury a large surface area of typically 1500 to 10 000 Å^2^ 16,19. The total contact area of the interface formed by the zinc hook domain of Rad50 protein is only 640 Å^2^ 6,20, suggesting a major effect from a low number of interactions and a significant contribution of intermolecular Zn^2+^ binding to the stability of the interaction.

Based on the currently available structure of the zinc hook domain of Rad50 from *P. furiosus* (PDB ID: 1L8D)^6^ (Fig. 1a), we designed a series of peptides of various lengths ranging from the CXXC motif required for intermolecular Zn^2+^ binding to the full-length domain. We also used alanine substitutions and the replacement of the amide bond with an ester bond to address the influence of certain residues and hydrogen bond formation in the peptide backbone on zinc hook complex stability. Using these models, we dissected the structural and thermodynamic determinants governing the stability of the metal-mediated dimer assembly formed by the zinc hook domain.

## Results and Discussion

**Figure 1:**
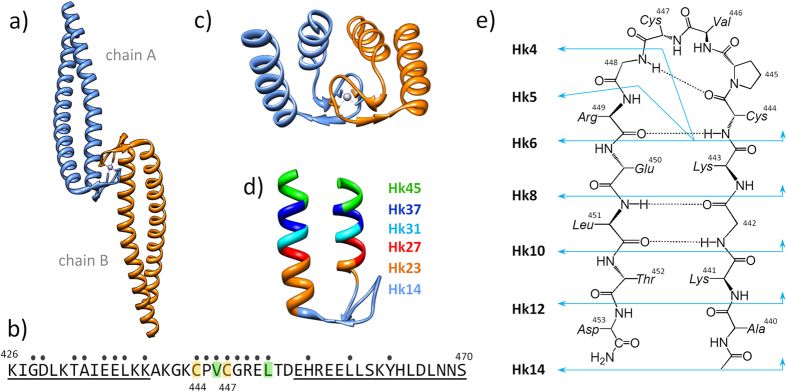
Structure of the central fragment of the Rad50 protein from *P. furiosus.* (**a**) Crystal structure of the 103-amino acid-long central fragment of Rad50, representing the zinc hook domain (PDB code: 1L8D)^6^; (**b**) Sequence of the 45-amino acid Hk45 protein fragment used as a domain model in the present study. The dots indicate all residues forming the dimer interface, obtained using the PDBePISA web server. Val446 and Leu451 (green) residues were examined for the formation of hydrophobic interactions^19^. Cys444 and Cys447, which participate in Zn^2+^ binding, are shown in yellow. The underlined residues participate in coiled-coil formation; (**c**) Structural representation of the 45-amino acid-long fragment of the domain; (**d**) Zinc hook peptides (Hk23-Hk45) used in the present study to probe the effect of coiled-coil formation; and (**e**) Zinc hook peptides (Hk4-Hk14) used in the present study to probe the effect of β-hairpin formation.

Based on the previous studies^6^ demonstrating the absolute requirement of Zn^2+^ for formation of the Rad50 dimer, we envisioned the formation of the complex as a reaction between two monomers and the Zn^2+^ ion to form a homodimeric zinc hook complex, Zn(Hk)_2_. For the structural and thermodynamic analysis of the zinc hook domain, we designed a series of zinc hook (Hk) peptides ranging in length from 4 to 45 amino acids (Hk4-Hk45). The 45-amino acid zinc hook domain peptide model (Hk45) used in the present study includes all residues that form intermolecular contacts in the complex and contains both the α-helical and β-hairpin regions of the domain (Fig. 1b,c)^20^. To examine the effect of the helical region on the Zn^2+^ binding thermodynamics, we shortened the helical region from both the N- and C-termini to 37 (Hk37), 31 (Hk31), 27 (Hk27) and 23 (Hk23) amino acid residues (Fig. 1d). Similarly, we designed a series of peptides (Hk4-Hk14) to investigate the structural and energetic effects of the formation of the central β-hairpin in the zinc hook structure (Fig. 1e).

### Zn^2+^-coupled folding of the hook domain

**Figure 2:**
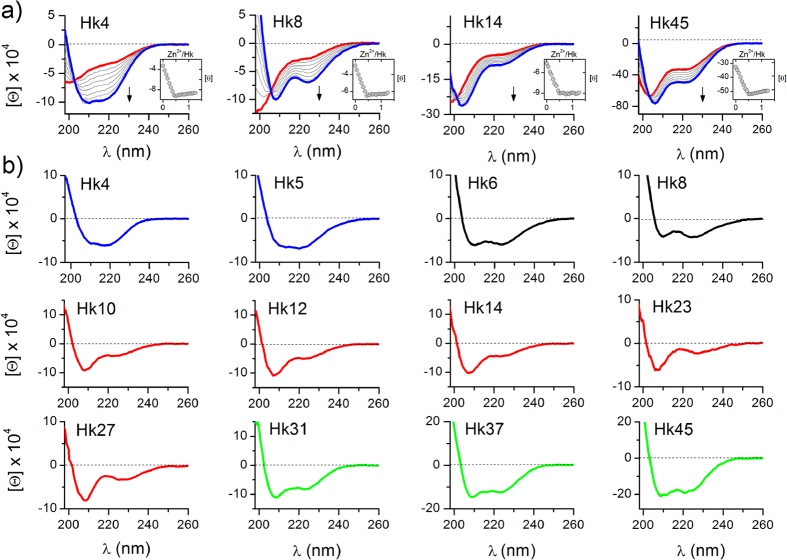
CD spectra of zinc hook peptides recorded in 10 mM Tris-HCl buffer, 100 mM NaClO_4_, 100–200 μM TCEP, pH 7.4. (**a**) Titrations of Hk4, Hk8, Hk14, and Hk45 with Zn^2+^ ions. The insets show the dependence of ellipticity at 220 nm on the Zn^2+^/Hk molar ratio. Red and blue colors refer to spectra of metal-free hook peptides and Zn(Hk)_2_ complexes, respectively; (**b**) Differential CD spectra of the zinc hook peptides obtained by the subtraction of spectra of free peptides from the spectra of Zn(Hk)_2_ complexes.

The binding of Zn^2+^ ions to hook peptides was monitored by circular dichroism spectroscopy (CD). Far-UV CD titrations, performed by the addition of Zn^2+^ to the initially metal-free peptide at pH 7.4 in the presence of TCEP (non-metal binding reductant)^21^, demonstrated extensive conformational changes upon Zn^2+^ binding. As expected, all hook peptides preferentially form Zn^2+^-mediated dimers (Zn(Hk)_2_) at pH 7.4, as indicated by a sharp inflection in the titration curves at a 1:2 Zn^2+^-to-peptide molar ratio (insets of Fig. 2a and Fig. S1). Depending on the examined peptide, the Zn(Hk)_2_ complexes adopt different conformations, as indicated by the distinct CD spectra obtained (blue curves, Fig. 2a and Fig. S1). The structural changes occurring during the association of hook subunits into Zn(Hk)_2_ can be compared based on the differential spectra obtained through the spectral subtraction of the hook peptides from their Zn^2+^ complexes (Fig. 2b). The differential spectra of the Hk4-Hk14 series showed features characteristic for the CD spectra of β-rich proteins and isolated type II β-turns^22^. The differential CD spectra of the Hk23-Hk27 series were similar to those of the Hk10-Hk14 series, likely reflecting the fact that the helical fragments of the domains in Hk23 and Hk27 are too short to form the stable helical/coiled-coil structure observed in the crystal structure. For the Hk31-Hk45 series, as the peptide length increased, a gradual increase in negative ellipticity and an increase in the ratio of ellipticities at 208 and 222 nm were observed, indicating an increase in the helical and coiled-coil content in the structure^23^. This increase in Zn^2+^-dependent structural changes with peptide length indicates that Zn^2+^ binding is coupled to the global folding of the domain, which potentially propagates to the ~500 Å distant globular domain of Rad50^13^. This interpretation is consistent with previous findings showing that the disruption of Zn^2+^ binding to the hook domain results in the complete loss of Rad50 activity^6^,^24^ and that mutations affecting the structure of the zinc hook influence functions in the globular domain of Rad50^11^,^13^. Interestingly, quite similar Zn^2+^-coupled folding associated with cooperative formation of a helical motif was observed for model peptides of a treble-clef zinc finger^25^.

### Stability of the zinc hook domain

We used potentiometric (Hk4-Hk14) and competitive CD titrations with zinc chelators (Hk23-Hk45) to measure the stability of the zinc hook complexes (Tables 1 and S1, Fig. S3 and Fig. S4)^15^,^26^. Both potentiometric and CD titrations of Hk peptides with Zn^2+^ indicated that Zn^2+^-mediated dimers (Zn(Hk)_2_) are formed preferentially at pH 7.4 (Fig. 2 and Fig. S3). The cumulative constant (*K*_12_, also known as *β*_2_) for the formation of the dimeric Zn(Hk)_2_ complex is given by equation (1):


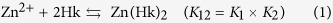


The stepwise constants *K*_1_ and *K*_2_ refer to the formation of the complexes one step at a time as noted in equations (2) and (3):


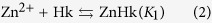



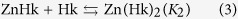


**Figure 3:**
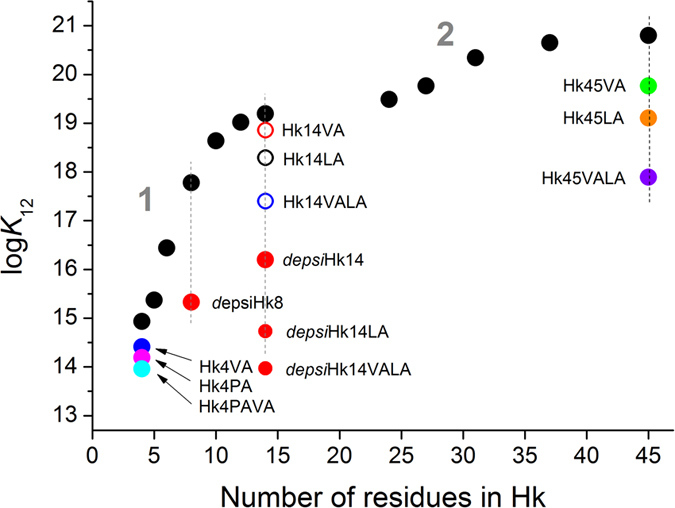
Relationship between the apparent formation constants (log*K*_12_) of the Zn(Hk)_2_ complex at pH 7.4 and the length of the zinc hook peptide. Black circles correspond to Hk4-Hk45 with native sequences. The *K*_12_ values for Hk14VA, Hk14LA and Hk14VALA mutants were adopted from the previous study^27^. Numbers 1 and 2 refer to two ranges of stability increase as discussed in the text.

The apparent formation constants (log*K*_12_) of the dimeric Zn(Hk)_2_ complexes at pH 7.4, calculated either from protonation and stability constants determined potentiometrically (see example in Supplementary information) or from CD competition experiments with zinc chelators, are presented in Table 1. It should be noted that the formation constant of Zn(Hk14)_2_ determined in this work is consistent with previously published data determined using a fluorescently labeled hook peptide of the same sequence^27^. A comparison between these constants and the length of the peptide presented in Fig. 3 revealed two ranges where relatively small changes in the peptide length resulted in a substantial difference in complex stability. The largest increase in the stabilization of dimeric species, ~4.3 orders of magnitude, was observed between Hk5 and Hk14, corresponding to the β-hairpin structure, whereas an additional increase in affinity, by ~1.5 orders of magnitude, was observed between Hk27 and Hk45, associated with an increase in the formation of the helical and coiled-coil structures observed in these peptide complexes (Fig. 2b). Similar dependence of the stability of zinc finger-like complexes on the length of peptide chain has been observed in previous studies, although in those works electrostatic or hydrophobic head-to-head or head-to-tail interactions were indicated as the reason for elevated stability^25^,^28^,^29^.

### Formation of the hydrogen bond network induced by Zn^2+^ binding

**Figure 4:**
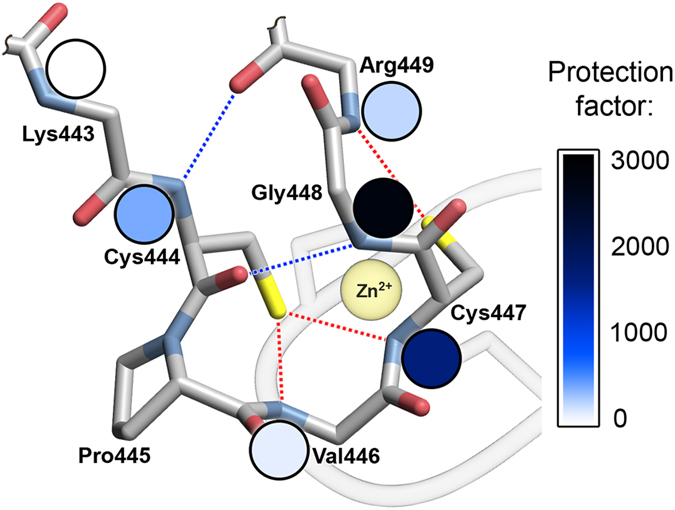
Backbone structure of the β-hairpin-forming fragment of the zinc hook domain. The colors of the circles correspond to the protection factors obtained for Zn(Hk10)_2_ in NMR deuterium exchange experiments. Blue and red dashed lines represent N–H···O and N–H···S hydrogen bonds, respectively. Details are presented in Tables S4 and S5.

Because the largest increase in the stabilization of dimeric zinc hook complexes occurred between the Hk5 and Hk14, we attempted to identify the structural features of these peptides and Zn^2+^ complexes. To this end, we examined the Hk4-Hk14 peptides in both metal-free and Zn^2+^ complexes using NMR and HDX MS. The 2D TOCSY, ROESY^1^, H/^13^C HSQC and ^1^H/^15^N HSQC analyses facilitated the nearly complete resonance assignment of the ^1^H^13^, C, and ^15^N chemical shifts for the Zn^2+^ complexes of the studied zinc hook peptides (Tables S2 and S3). Analysis of the 2D ROESY spectrum of the Zn(Hk14)_2_ complex generated 131 through-space contacts between proton pairs. A comparison of the experimental spectrum for Zn(Hk14)_2_ with a simulated spectrum for the corresponding fragment of the crystal structure (PDB ID: IL8D^6^) revealed no differences in the central part of the β-hairpin, while terminal residues displayed higher mobility (and fewer ROE contacts) in the peptide compared to their counterparts in the crystal structure. These data showed that the structure of the Zn(Hk14)_2_ complex observed in the solution is similar to the conformation of the corresponding fragment of the hook crystal structure. Using NMR, we measured the exchange rates and temperature coefficients of amide protons in the zinc complexes of Hk6, Hk8, Hk10 and Hk14. Amide proton exchange is a powerful method to probe structural and dynamic properties at residue-level resolution, facilitating the distinction of solvent-exposed amides and amides within structured regions of the protein protected against exchange. The amide proton solvent accessibility is expressed as a protection factor representing the exchange rate expected for amide protons in unfolded peptides under a given condition, divided by the observed rate of exchange (Table S4). It can be safely assumed that amide protons cannot be buried deeply in the protein structure of a short peptide; therefore a low temperature coefficient and high protection factor of amide proton are indicative of involvement in the formation of a hydrogen bond^30^,^31^. The NMR results showed that amides of Cys444, Cys447, Gly448 and Arg449 are highly protected from the solvent in Zn(Hk14)_2_ (Table S5), but no amides are protected from solvent exchange for the metal-free Hk14, evidenced by the 1D ^1^H NMR spectra (Fig. S5). The same pattern of protected amides was also observed for Zn(Hk10)_2_ but was less manifested in the Zn(Hk8)_2_ and Zn(Hk6)_2_. The structural mapping of these highly protected amides supports a network of N–H···O and N–H···S hydrogen bonds within the β-hairpin formed upon Zn^2+^ complexation (Fig. 4). These data showed that Zn^2+^ binding nucleates folding of the β-hairpin of the hook domain. This mechanism was also supported by the results of HDX MS of Zn^2+^ complexes of Hk4-Hk14, which showed three protected amide protons in the Zn^2+^ complexes of Hk10-Hk14: one amide proton was identified within Cys444, while another two amide protons were detected in the Gly448-Leu451 fragment and two protected protons were observed in the Hk6 complex, localized on Cys444 and either Gly448 or Arg449 (see Supplementary information for details, Figs S6–S10).

### Impact of β-hairpin formation on stability of the hooked structure

The increasing stability of the zinc hook complex with peptide length observed in Hk5-Hk14 supports the idea that the observed increase in stability is associated with the formation of the β-hairpin. Therefore we decided to investigate the isolated effect of the formation of the β-hairpin on the stability of the Zn(Hk)_2_ complexes. Based on previous results showing that β-hairpin folding begins with turn formation and propagates toward the tail^32^,^33^ and the metal-mediated folding of ββα zinc fingers is initiated with the formation of the turn that nucleates the β-hairpin^34^, we hypothesized that the disruption of the turn region will prevent formation of the β-hairpin structure^35^,^36^. To this end, the amide of Gly448 was replaced with an ester linkage, resulting in a loss of the hydrogen-bond donor of the β-turn in *depsi*Hk peptides (Fig. 3). The resulting *depsi*Hk8 and *depsi*Hk14 peptides exhibited significantly smaller changes in molar ellipticity at 222 nm compared with Hk8 or Hk14 upon Zn^2+^ binding (Fig. S11). The stability of Zn(*depsi*Hk8)_2_ and Zn(*depsi*Hk14)_2_ was significantly decreased by ~2.5 and ~3 orders of magnitude in the *K*_12_ values, respectively, compared with the parent Zn(Hk8)_2_ and Zn(Hk14)_2_ complexes (Fig. 3, Tables 1 and S6). We combined an amide-to-ester substitution with the previously described alanine substitutions of conserved residues of the hydrophobic core (Hk14VA, Hk14LA, and Hk14VALA)^27^. The combined mutations of hydrophobic residues and amide-to-ester substitutions in the 14-amino acid fragment (*depsi*Hk14LA and *depsi*Hk14VALA) decreased the stability of the Zn(Hk)_2_ species to the level of the Zn^2+^ complex formed by Hk4 peptide. These data indicate the additive effect of amide-to-ester substitutions and mutations of hydrophobic residues on the stability of the zinc hook complex, showing that hydrophobic interactions and backbone hydrogen bonding are the major structural factors governing the high stability of the complex (Fig. 3, Tables 1 and S6). Furthermore, β-turn-favoring Pro445 and hydrophobic Val446 in the spacer between two Zn^2+^ binding cysteines showed stabilizing effects, even in the context of minimal Hk4, as seen in Hk4VA, Hk4PA, and Hk4PAVA (Fig. 3, Tables 1 and S6).

### Influence of the conserved hydrophobic core on formation of the coiled-coil

We examined the effect of conserved hydrophobic residues (Val446 and Leu451) on the conformation and stability of the 45-mer zinc hook model by constructing peptides with either single or double substitutions to alanine (Hk45VA, Hk45LA and Hk45VALA). Compared with the Hk45 peptide, Hk45VA and Hk45LA showed two- and ten-fold less pronounced structural changes, respectively, upon the formation of the corresponding Zn(Hk)_2_ complexes, evidenced by changes in the molar ellipticity at 222 nm (Fig. S12). The double-mutated Hk45VALA showed even smaller changes in molar ellipticity upon the formation of Zn(Hk)_2_, indicating a marginal degree of metal-coupled folding (Fig. S12). Mutations of Val446 and Leu451 significantly affected the stability of the Zn(Hk)_2_ complex (Fig. 3, Table 1). The 2.85 order of magnitude decrease in stability, in terms of the *K*_12_ values between Hk45 and Hk45VALA, was larger than that between Hk14 and Hk14VALA (1.79 orders of magnitude)^27^. These data indicate that the interface formed by the Val446 and Leu451 residues of the β-hairpin and the hydrophobic residues of the coiled-coil, observed in the crystal structure, is essential for metal-coupled folding and the stable assembly of the hook domain.

### Acid-base properties of cysteine thiols in CXXC motif

**Figure 5:**
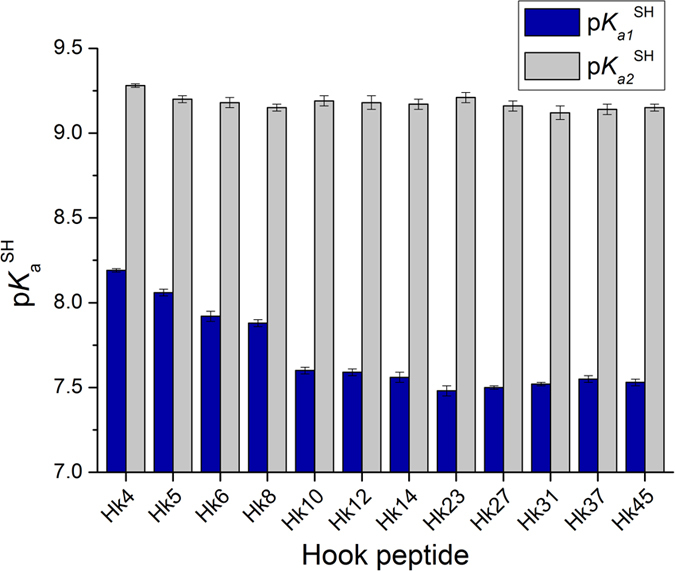


The acidity of the Zn^2+^ coordinating cysteines has a critical impact on the metal-binding properties as thiolate anions act as ligands to form complexes with transition metals^37^. The p*K*_a_ of a cysteine thiol (here p*K*_a_^SH^) can be strongly influenced by its protein microenvironment^38^,^39^. Thus, the deprotonation of cysteine thiols was spectrophotometrically measured at 220 nm using metal-free forms of the Hk peptides (Fig. 5)^15^,^40^,^41^. The obtained p*K*_a1_^SH^ and p*K*_a2_^SH^ values were consistent with the values obtained in potentiometric titrations (Table S7, Fig. S13). Interestingly, a significant increase in the acidity of one cysteine thiol (p*K*_a1_^SH^ = 8.2 − 7.5) was observed as the peptide chain length increased from Hk4 to Hk10, whereas the p*K*_a2_^SH^ of a second cysteine thiol remained constant at ~9.2. Further increases in the domain length up to Hk45 did not result in additional increases in thiol acidity (Table S7). These data showed that the 10-amino acid-long central region of the hook hairpin (Hk10) in the metal-free form captures the essential structural features necessary to accommodate the significantly perturbed p*K*_a_ values of the thiol group of one of the cysteine residues in the hook CXXC motif, thereby reducing the enthalpic cost of thiol deprotonation associated with Zn^2+^ complex formation (see below). Interestingly, the CXXC motif in the active site of certain enzymes, such as the thiol-disulfide oxidoreductases of the thioredoxin superfamily, is characterized by similarly decreased p*K*_a_^SH^ of the N-terminal cysteine compared with the second cysteine^39^,^42^. We postulate that the CXXC motif in hook peptides might adopt a similar conformation, suggesting that the reduced p*K*_a1_^SH^ value of ~7.5 corresponds to Cys444. It has been proposed that Zn^2+^ assisted deprotonation of all the cysteines during the folding of Zn(Cys)_4_ zinc-finger cores and that the ionized cysteine core is stabilized by the interactions with the protein-derived structural elements^25^,^43^. The formation of N–H···S hydrogen bonds, which we also observed in the zinc hook structure, has been proposed to be an important factor for such stabilization^38^. Such stabilization of the Zn(Cys)_4_ core by the protein-derived structural elements is expected to results in both stabilization of the Zn^2+^ complex and increase in the apparent acidity of the peptide thiols in the presence of Zn^2+^. To test this prediction, we performed spectrophotometric pH titrations of zinc hook peptides at a 1:2 Zn^2+^-to-peptide molar ratio. The results of these titrations were used to calculate the apparent average value of peptide thiols’ deprotonation in the presence of Zn^2+^ ions (p*K*_a_’), which reflects the competition between protons and Zn^2+^ for binding to cysteine thiolates^15^,^28^. The obtained p*K*_a_’ values (from 6.25 for Hk4 to 4.70 for Hk45, Table S8) and apparent formation constants (log*K*_12_) of the zinc complexes are highly correlated (Table 1, Fig. S14), which is consistent with the notion of the Zn^2+^-assisted deprotonation the cysteines, and stabilization of the Zn(Cys)_4_ core by the protein-derived structural elements. The p*K*_a_’ values of 5.1–5.6 were reported for the Zn^2+^-induced Cys deprotonation in the case of (Cys)_4_ and (Cys)_3_(His) zinc fingers^14^,^29^,^37^,^44^. Recently, the p*K*_a_’ value of cysteines between 4.2 and 5.1 was reported for the artificial (Cys)_4_ treble-clef zinc finger L_TC_, but the exact deprotonation value was not calculated^25^. It should be noted that the p*K*_a_’ values obtained in different studies cannot be directly compared, because p*K*_a_’ values are specific for particular conditions (i.e. concentrations and Zn^2+^-to-peptide ratios).

### Thermodynamics of zinc hook domain formation

**Figure 6:**
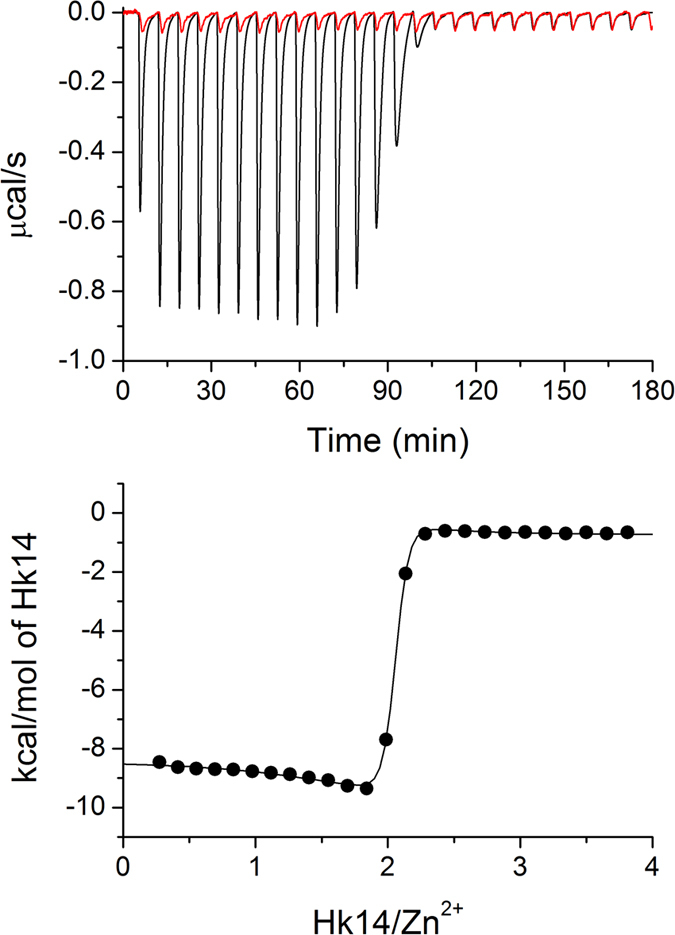
ITC titration of Zn^2+^ with Hk14 peptide. Upper panel shows calorimetric response (raw injection heats) of 50 μM Zn^2+^ titrated with 1.3 mM Hk14 peptide in 50 mM HEPES, pH 7.4, 25 °C (black trace) and blank titration with 1.3 mM Hk14 peptide into buffer (red trace). Lower panel displays the corresponding calorimetric binding isotherm.

In order to study the thermodynamics of Zn^2+^ binding to Hk4-Hk45 peptides in detail, we used ITC to determine the enthalpies of formation of the corresponding Zn(Hk)_2_ complexes (Fig. 6 and Fig. S15). The resulting experimental enthalpies (Δ*H*_ITC_) of the formation of Zn(Hk)_2_ complexes are presented in Table 2, and the complete fit values are presented in Table S9. It should be noted that same enthalpies of the formation of Zn(Hk)_2_ were obtained by both titrations of Zn^2+^ ions into peptide and titrations of peptide into Zn^2+^ (Figs S16 and S17). Because Zn^2+^ complexation by cysteine-containing peptides is accompanied by thiol deprotonation, the experimental enthalpy (Δ*H*_ITC_) must be corrected for the heat of protonation of the buffer^45^,^46^, as indicated in equation (4):


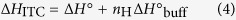


where Δ*H*° is the buffer independent intrinsic reaction enthalpy, *n*_H_ is the number of protons released over the course of the reaction, and Δ*H*°_buff_ is the buffer-specific heat of protonation (−5.02 kcal/mol for HEPES)^47^. The number of released protons upon Zn^2+^ binding is the number of protons associated with cysteine thiols of two metal-free peptide molecules at pH 7.4, calculated based on the p*K*_a1_^SH^ and p*K*_a2_^SH^ values (Table S7)^48^. The resulting buffer-independent intrinsic reaction enthalpy, Δ*H*°, can be further dissected into two components: the enthalpy of Zn^2+^ binding to the peptide and the associated structural changes (Δ*H*°_Zn−pep_) and enthalpy of deprotonation of cysteine thiols associated with Zn^2+^ binding (*n*_H_Δ*H*°_CysH_)^14^, as expressed in equation (5):


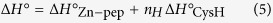


where *n*_H_ is the number of protons released over the course of the reaction (see above), and Δ*H*°_CysH_ is the heat of Cys deprotonation. The average value of the last parameter is +8.5 kcal/mol^49^, and it is assumed that it is constant in the studied series of peptides. In addition to these factors, the entropic component of the reaction (−TΔ*S*°) was calculated based on Gibbs’ law, as shown in equation (6):


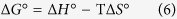


This equation uses the Δ*G*° values that were calculated based on the apparent formation constants (*K*_12_) of the hook complexes from Table 1 as indicated in equation (7):


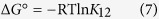


**Figure 7:**
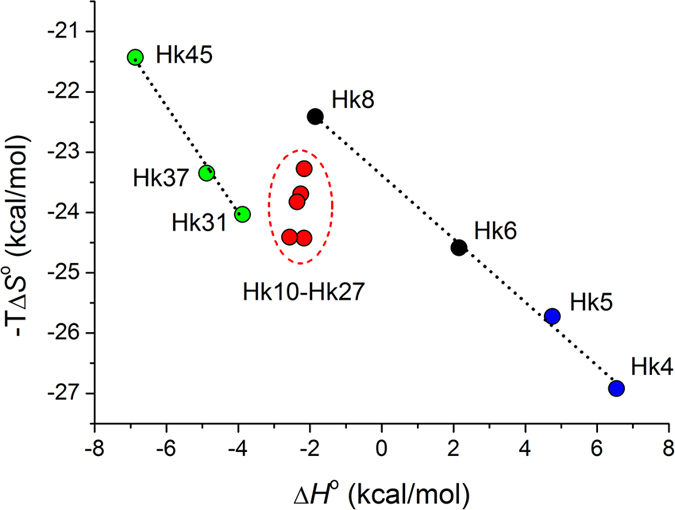
Entropy-enthalpy relationships of the zinc hook complexes. Entropy-enthalpy compensation (EEC) is observed for peptide series where an increase in peptide length increases the folded structure in the corresponding zinc hook complexes (Hk4-Hk8 and Hk31-Hk45) but not for peptide series that present a similar number of folded structures in the dimeric zinc hook complex, regardless of length (Hk10-Hk27).

**Figure 8:**
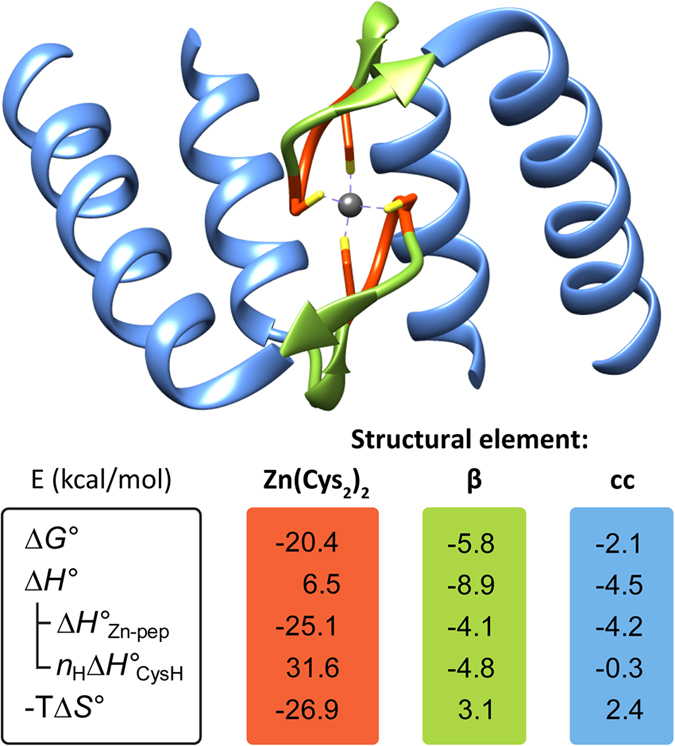
Thermodynamic and structural contributions to the stability of the zinc hook dimer. The values should be read according to the formula Δ*G*° = Δ*H*°_Zn-pep_ + *n*_H_Δ*H*°_CysH_ − TΔ*S*°. Zn(Cys_2_)_2_, β, and cc refer to the thermodynamic contributions in kcal/mol to the complex formation by the particular structural component of the zinc hook domain: Zn^2+^ binding motif, β-hairpin and coiled-coil, respectively.

A comparison of the Δ*G*°, Δ*H*°, and −TΔ*S*° listed in Table 2 revealed that the formation of the zinc hook complexes is largely entropically driven. The entropically driven binding of Zn^2+^ has been demonstrated to be likely an intrinsic property of Zn(Cys)_4_ coordination spheres in proteins^14^, whereas Zn^2+^ binding to the sites containing histidine ligands are more enthalpically (and less entropically) driven as the number of histidine ligands increases^48^,^50^. The increase in stability of the hook complexes with the peptide length, reflected as the decrease of the Δ*G*° values, results primarily from the increase in favorable enthalpy (Δ*H*°). The observed dependence of the enthalpy on the domain fragment length is partially compensated by the unfavorable change in entropy (−TΔ*S*°), demonstrating entropy-enthalpy compensation (EEC)^51^. As evident in Fig. 7, EEC was only observed in the series of peptides where increasing peptide length paralleled an increasing tendency for secondary structure formation resulting from Zn^2+^ binding (Fig. 2b): β-hairpin in Hk4-Hk8 and coiled-coil in Hk31-Hk45. For these peptide series, the enthalpy of binding becomes more favorable, and the entropy becomes more unfavorable, as the length of the structured fragment increases, suggesting that the favorable increase in enthalpy is closely correlated with the interactions resulting from metal-mediated folding; however, this increase is partially compensated by the unfavorable loss in entropy resulting from the formation of ordered structures and the association of the subunits. Next, we analyzed in detail the thermodynamic contributions from the structural components present in the hook domain to the stability of the hook complex. The minimal hook peptide Hk4 can serve as a reference for the Zn(Cys_2_)_2_ zinc hook coordination motif in the absence of other protein-derived structural elements (Fig. 7). The Δ*H*°_Zn-pep_ value for Zn(Hk4)_2_ (−25 kcal/mol) was identical to that obtained for the Gly-rich peptide previously used to model the Zn(Cys)_4_ coordination motif of structural zinc sites, such as those found in zinc fingers, in the absence of protein-derived structural elements, indicating that the enthalpy of Zn^2+^–S bond formation is similar in these two models^14^,^37^. In order to obtain information on the thermodynamic contributions from the structural components present in the hook domain, we compared the thermodynamic parameters (Δ*G*°, Δ*H*°, −TΔ*S*°, Δ*H*°_Zn-pep_, *n*_H_Δ*H*°_CysH_) for the formation of Zn(Hk)_2_ complexes of the peptides that encompass particular structural fragments (Table 2). Subtraction of the thermodynamic parameters (Δ*G*°, Δ*H*°, −TΔ*S*°, Δ*H*°_Zn-pep_, *n*_H_Δ*H*°_CysH_) for Zn(Hk4)_2_ from the corresponding values for Zn(Hk14)_2_ and subtraction of the thermodynamic parameters for Zn(Hk14)_2_ from the corresponding values for Zn(Hk45)_2_ gave the information of the net thermodynamic effect of the protein-derived structural elements in the β-hairpin (β) and coiled-coil (cc) domain fragments respectively (Fig. 8). These analyses show that the formation of the Zn(Cys_2_)_2_ coordination motif (minimally as Zn(Hk4)_2_) provides −20.4 kcal/mol of Gibbs free energy, while protein-derived structural elements in the β-hairpin and the coiled-coil regions provide an additional −5.8 kcal/mol and −2.1 kcal/mol of free energy, respectively. The change in free energy emerges from favorable change in enthalpy (Δ*H*°) of −8.9 kcal/mol and −4.5 kcal/mol and an unfavorable entropic cost (−TΔ*S*°) of 3.1 kcal/mol and 2.4 kcal/mol for β-hairpin and coiled-coil regions respectively. The analysis of the enthalpic component (Δ*H*°) according to equation (5) shows that the β-hairpin formation contributes −4.1 kcal/mol to the enthalpy due to the favorable change in enthalpy of Zn^2+^ binding and the associated structural changes (ΔΔ*H*°_Zn-pep_) and −4.8 kcal/mol due to the decrease in the energetic cost of cysteine deprotonation (Δ(*n*_H_Δ*H*°_CysH_)). Correspondingly, the coiled-coil fragment contributes −4.2 kcal/mol to the favorable change in enthalpy of Zn^2+^ binding and the associated structural changes (ΔΔ*H*°_Zn-pep_), providing only a negligible decrease in the energetic cost of cysteine deprotonation (Δ(*n*_H_Δ*H*°_CysH_)) (Fig. 8). This energetic effect is directly associated with the increased acidity of one of the thiols in the metal-free form, occurring almost entirely in the β-hairpin-forming fragment (Fig. 5). These data show that the contribution to the stability of the β-hairpin-forming fragment is evident in both metal-free and Zn^2+^-bound forms. In the metal-free form, an increase of the acidity of one of the thiols decreases the unfavorable enthalpy of deprotonation, whereas in the Zn^2+^ complex, a network of hydrogen bonds and other interactions is formed, increasing the favorable enthalpy of the reaction.

### Comparison with the stability of other zinc binding domains

Currently, very limited data on metal ion affinity for intermolecular Zn^2+^ binding domains exist. One example of a relatively well-studied domain is the intermolecular zinc binding site formed by CD4/CD8α co-receptors and Lck kinase. The apparent formation constants (log*K*) were reported as 6.4 for CD4-Lck and 6.05 for CD8α-Lck^5^. However, these values were measured in the presence of excess Zn^2+^, and thus the metal concentration factor was neglected in the calculation of the stability constant. In the following study, the authors considered both the metal and peptide concentrations and measured the apparent stability constants of Co^2+^-mediated dimers formed by minimal peptides from CD4, CD8α and Lck reporting values, which expressed as apparent formation constants (log*K*) are 7.8 for the CD4-Co^2+^-Lck complex and 8.1 for the CD8α-Co^2+^-Lck complex^52^.

In contrast to intermolecular domains, intramolecular zinc binding domains, such as zinc fingers, have been studied in considerable detail in terms of their metal-binding affinity. The typical values of log*K* reported for natural zinc binding fingers ranges from 10 to 14^1^,^14^,^15^,^29^,^53^,^54^. The stability values reported for intramolecular (ZnL) complexes are not directly comparable with the constants of dimeric complexes (ZnL_2_) presented here (Table 1) because of the different definitions of the equilibrium constants. An indirect way to compare these conditional equilibrium constants is to analyze relative complex formation in a setting where both peptides compete for Zn^2+^ using e.g. Hyperquad Simulation and Speciation software^55^. Our analysis revealed that the zinc hook domain would thermodynamically outcompete the typical or even the strongest natural zinc finger domains (Fig. S18). These results show that zinc hook and other similar intermolecular Zn^2+^ binding sites in proteins can form metal-mediated assemblies at very low concentrations of subunits under physiologically buffered concentrations of Zn^2+^ ions^56^.

### Conclusions

The present study provides a detailed analysis of the determinants of intermolecular Rad50 zinc hook domain stabilization using model peptides of gradually increasing lengths and mutational variants. Although we anticipated that the Zn^2+^ affinity would increase with hook peptide length, thereby increasing the total number of intra- and inter-molecular interactions, most of the stabilization effect reflected a small portion of the Rad50 hook domain surrounding the Zn^2+^ binding motif. The significant stabilization of the hook complex resulted from the favorable increase in the enthalpy of Zn^2+^ complexation. This reflects a reduction of the unfavorable enthalpy of Cys thiol deprotonation ascribed to the β-hairpin forming fragment and the favorable enthalpy of interactions in the β-hairpin and coiled-coil structures of the domain, which are formed upon metal binding. We postulated that this metal-coupled folding dictates molecular recognition and the specificity and stability of the interaction and is responsible for the long-range allostery observed between the hook and globular domains of Rad50 in recent studies^11^,^13^,^23^.

## Methods

### Peptide synthesis

Zinc hook peptides (Hk) were synthesized via solid-phase synthesis (SPPS) using an Fmoc strategy. All peptides were N-terminally acetylated. Amide-to-ester backbone bond-substituted peptide analogs (*depsi*Hk) were synthesized according to Jemth and co-workers, followed by fragment condensation^35^.

### Protonation and Zn^2+^ stability constants

The protonation and Zn^2+^ stability constants of Hk4-Hk14 zinc hook peptides and depsipeptides were determined in potentiometric titrations. The apparent formation constants of Zn^2+^ complexes with zinc hook peptides (Hk23–45, Hk45VA, Hk45LA, and ZnHK45VALA) were determined in the spectropolarimetric titrations with Zn^2+^ in the presence of HEDTA, EDTA and TPEN chelators^15^,^27^. The p*K*_*a*1_^SH^, p*K*_*a*2_^SH^ dissociation constants of the cysteine thiols of Hk4-Hk45 were determined spectrophotometrically as described previously^41^. Similarly, the dissociation constants of thiols in the presence of Zn^2+^ (p*K*_a_’) were determined by the pH titration of Hk4-Hk14 and Hk45 in the presence of 0.495 eq. of Zn^2+^.

### NMR studies

NMR measurements of 5 mM Zn(Hk6)_2_, Zn(Hk10)_2_, Zn(Hk12)_2_ and Zn(Hk14)_2_ and metal-free Hk14 were performed in degassed 10% D_2_O in H_2_O (pH 7.4) on a DDR2 Agilent 600 MHz spectrometer equipped with a Penta probe.

### Hydrogen-deuterium exchange mass spectrometry (HDX MS)

The experiments were conducted in the exchange-in mode, e.g., hydrogen into deuterium. For each deuterated sample, isotopic profiles of 1:1 and 1:2 metal-to-peptide complexes were compared with the theoretical isotopic profile of fully deuterated species, and the signals corresponding to complexes with various numbers of protected protons were analyzed. Subsequently, the fragmentation ions were analyzed in the same manner.

### Isothermal titration calorimetry (ITC)

The binding of Zn^2+^ to Hk peptides was monitored using ITC at 25 °C. All experiments were performed in HEPES buffer (*I* = 0.1 M from NaCl) at pH 7.4 under an argon atmosphere. The Hk peptide (titrant) concentration was 1.3 mM, whereas the metal (titrate) concentration was 50 μM. The titration data were fitted to a binding model accounting for the formation of ZnHk and Zn(Hk)_2_ complexes during the course of titration.

For a more detailed description of the experimental methods of peptide synthesis and zinc hook complex characterization (NMR, ITC, HDX MS, UV-vis, and circular dichroism spectroscopy), see Supplementary Information.

## Additional Information

**How to cite this article**: Kochańczyk, T. *et al*. Metal-coupled folding as the driving force for the extreme stability of Rad50 zinc hook dimer assembly. *Sci. Rep.* **6**, 36346; doi: 10.1038/srep36346 (2016).

**Publisher’s note:** Springer Nature remains neutral with regard to jurisdictional claims in published maps and institutional affiliations.

## Supplementary Material

Supplementary Information

## Figures and Tables

**Table 1:** Apparent formation constants (log*K*_12_) of Zn(Hk)_2_ complexes of zinc hook peptides and their mutants at pH 7.4, *I* = 0.1 M, 25 °C.

Hook peptide	log*K*_12_	Mutant peptide	log*K*_12_
Hk4	14.93 ± 0.02	Hk4VA	14.41 ± 0.02
Hk5	15.37 ± 0.02	Hk4PA	14.19 ± 0.02
Hk6	16.44 ± 0.02	Hk4PAVA	13.96 ± 0.02
Hk8	17.78 ± 0.01	Hk45LA	19.06 ± 0.08
Hk10	18.64 ± 0.01	Hk45VA	19.78 ± 0.08
Hk12	19.02 ± 0.01	Hk45LAVA	17.89 ± 0.08
Hk14	19.19 ± 0.01		
Hk23	19.49 ± 0.04	Depsipeptide	log*K*_12_
Hk27	19.77 ± 0.04	*depsi*Hk8	15.33 ± 0.01
Hk31	20.47 ± 0.09	*depsi*Hk14	16.21 ± 0.01
Hk37	20.69 ± 0.06	*depsi*Hk14LA	14.73 ± 0.03
Hk45	20.74 ± 0.06	*depsi*Hk14VALA	13.97 ± 0.02

Values were calculated from cumulative protonation and stability constants determined potentiometrically (Tables S1 and S6) or determined spectropolarimetrically in competition experiments with zinc chelators (Fig. S4) and are not the results of ITC data fits.

**Table 2:** Thermodynamic parameters of Zn(Hk)_2_ complex formation at pH 7.4, *I* = 0.1 M, 25 °C.

Zinc hook peptide	Δ*G*° (kcal/mol)	Δ*H*_ITC_ (kcal/mol)	Δ*H*° (kcal/mol)	Δ*H*°_Zn-pep_ (kcal/mol)	n_H_Δ*H*°_CysH_ (kcal/mol)	−TΔ*S*° (kcal/mol)
Hk4	−20.37 ± 0.03	−12.1 ± 0.03	6.54	−25.08	31.62	−26.92
Hk5	−20.97 ± 0.03	−13.5 ± 0.02	4.75	−26.19	30.94	−25.73
Hk6	−22.43 ± 0.03	−15.5 ± 0.02	2.15	−27.77	29.92	−24.58
Hk8	−24.26 ± 0.01	−19.3 ± 0.02	−1.85	−31.43	29.58	−22.41
Hk10	−25.43 ± 0.01	−18.2 ± 0.02	−2.16	−29.36	27.2	−23.28
Hk12	−25.95 ± 0.01	−18.3 ± 0.02	−2.26	−29.46	27.2	−23.70
Hk14	−26.18 ± 0.01	−18.2 ± 0.02	−2.36	−29.22	26.86	−23.82
Hk23	−26.59 ± 0.05	−17.7 ± 0.03	−2.17	−28.52	26.35	−24.43
Hk27	−26.97 ± 0.05	−18.1 ± 0.03	−2.57	−28.92	26.35	−24.41
Hk31	−27.9 ± 0.01	−19.5 ± 0.03	−3.88	−30.40	26.52	−24.03
Hk37	−28.22 ± 0.08	−20.6 ± 0.04	−4.88	−31.57	26.69	−23.35
Hk45	−28.29 ± 0.08	−22.5 ± 0.03	−6.87	−33.39	26.52	−21.43

The Δ*G*° values were calculated based on apparent formation constants *K*_12_ presented in Table 1 using equation (7).
