# Supplementary material for: Metal-coupled folding as the driving force for the extreme stability of Rad50 zinc hook dimer assembly
Source: Sci Rep. 2016 Nov 3;6:36346. doi: 10.1038/srep36346 (PMC5093744; doi:10.1038/srep36346)
Supplement: Supplementary Information [file srep36346-s1.pdf]

Supplementary information

Metal-coupled folding as the driving force for the extreme stability of Rad50 zinc hook dimer assembly

Tomasz Kochańczyk[1], Michał Nowakowski[2], Dominika Wojewska[1], Anna Kocyła[1], Andrzej Ejchart[3], Wiktor Koźmiński[2] and Artur Krężel[1]*

Contribution from

[1]*Department of Chemical Biology, Faculty of Biotechnology, University of Wrocław, Joliot-Curie 14a, 50-383 Wrocław, Poland,*

[2]*Faculty of Chemistry, Biological and Chemical Research Centre, University of Warsaw, Żwirki i Wigury 101, 02-089 Warsaw, Poland,*

[3]*Institute of Biochemistry and Biophysics, Polish Academy of Science, Pawińskiego 5a, 02-106 Warsaw, Poland*

Correspondence to Artur Krężel,

E-mail: artur.krezel@uwr.edu.pl

Experimental section

Peptide synthesis

Zinc hook peptides (Hk) were synthesized via solid-phase synthesis (SPPS) using an Fmoc-strategy on a TentaGel R RAM Amide Rink (Rapp Polymere GmbH, Tuebingen Germany) resin (substitution 0.2 mmol/g) and a Liberty 1 microwave-assisted synthesizer (CEM). All peptides were N-terminally acetylated. Amide-to-ester backbone bond-substituted peptide analogs (*depsi*Hk) were synthesized according to the published procedure[1] followed by fragment condensation. The crude peptides were purified via high-performance liquid chromatography (HPLC) (Dionex Ultimate 3000) on Phenomenex PEPTIDE XB-C18 columns using 0.1% TFA in H_2O with an acetonitrile (ACN) gradient. The purified peptides were identified by electrospray ionization (ESI) mass spectrometry with an API 2000 Applied Biosystems instrument. The identified and calculated mass values are listed in Table S9.

Depsipeptide synthesis

Depsipeptides were synthesized via the fragment condensation of protected Ac-AKGKCPV-OH with resin-attached C*GRELTD (Scheme S1).[1] Briefly, the protected RELTD peptide was synthesized on a TentaGel R RAM Amide Rink. After Fmoc group removal from the arginine residue, the resin containing the peptide was mixed with 8 eq. of glycolic acid, 7 eq. of (1-cyano-2-ethoxy-2-oxoethylidenaminooxy)-dimethylamino-morpholino-carbenium hexafluorophosphate (COMU) and 16 eq. of *N*,*N*-diisopropylethylamine (DIEA) in DMF and then incubated for 5 min at 75°C in a Discovery microwave-assisted (CEM) reactor and for 1 h at room temperature. Subsequently, 5.6 eq. of Fmoc-Cys(Trt)-OH was dissolved in cold dichloromethane/*N*,*N*-dimethylformamide (DCM/DMF; 50/50 v/v), mixed with 5 eq. of *N,N'*-diisopropylcarbodiimide (DIC) and kept on ice for 15 minutes. Finally, this mixture was added to the resin, followed by the addition of 0.1 eq. of 4-dimethylaminopyridine (DMAP) and 2 eq. of N-ethyl-morpholine (NEM); the mixture was then incubated for 1 h at room temperature with agitation. The coupling step was repeated once. Ac-AKGKCPV-OH was synthesized on a 2-chlorotrityl chloride resin (100-200 mesh, 1% divinylbenzene [DVB]), according to the standard SPPS Fmoc-strategy. The acetylated peptide with side-chain protection groups was cleaved from the resin (Scheme S1). The final coupling step was performed in DCM/DMF (50/50 v/v) with the protected Ac-AKGKCPV peptide provided in 1.5-molar excess over resin-attached C*GRELTD peptide in the presence of 2 eq. of COMU

and 3 eq. of DIEA for 12 h. Other depsipeptides used in this study (*depsi*Hk8, *depsi*Hk14LA, and *depsi*Hk14VALA) were synthesized using the same procedure. The N-terminal-acetylated resin-attached peptides were cleaved from the resin using a mixture of trifluoroacetic acid (TFA)/thioanisole/1,2-ethanedithiol (EDT)/anisole (90/5/3/2 v/v/v/v) for 2 h. The crude peptides were purified via high-performance liquid chromatography (HPLC) (Dionex Ultimate 3000) on Phenomenex PEPTIDE XB-C18 columns using 0.1% TFA in H_2O with an acetonitrile (ACN) gradient. The purified peptides were identified by electrospray ionization (ESI) mass spectrometry with an API 2000 Applied Biosystems instrument. The identified and calculated mass values are listed in Table S10.

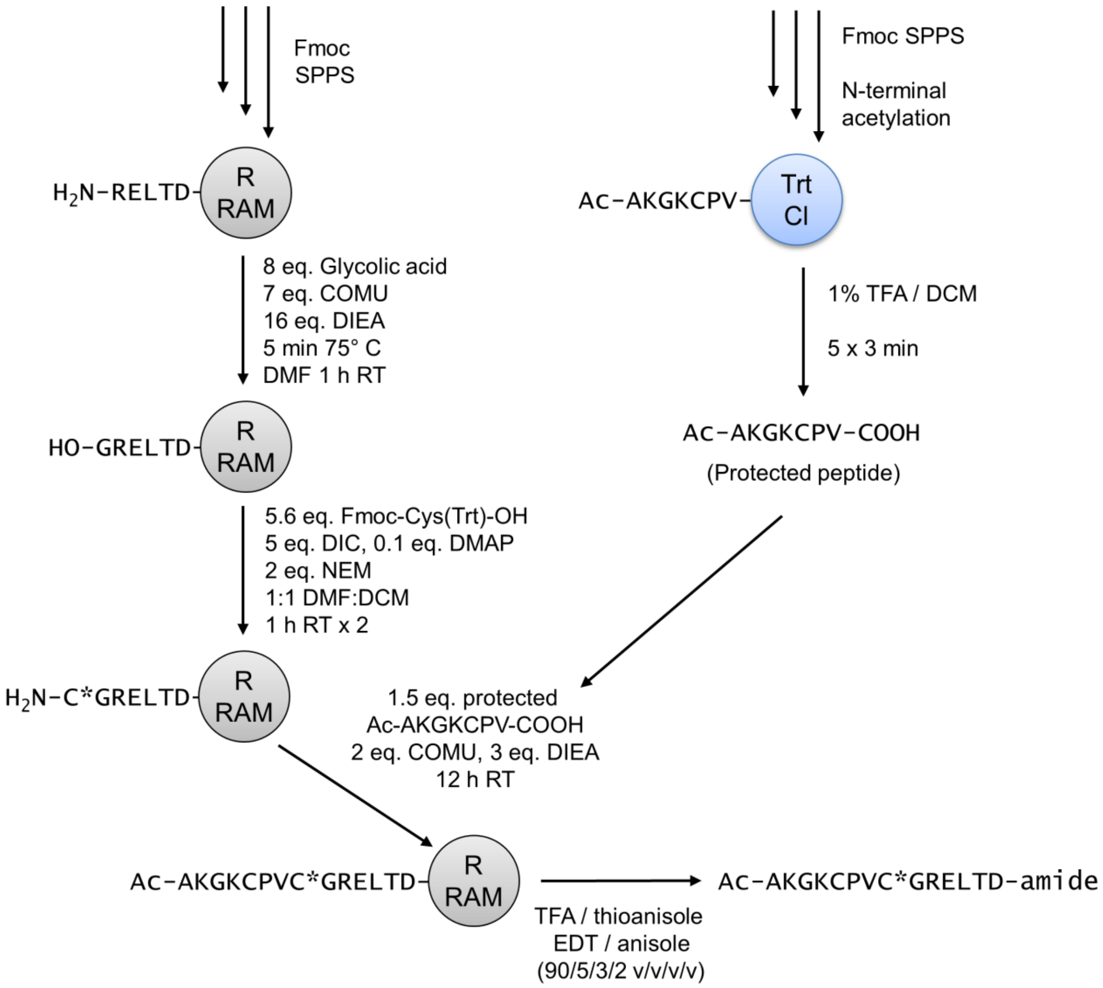

Scheme S1. Scheme of the synthesis of *depsi*Hk14 peptide. Asterisk (*) denotes localization of the ester bond.[1]

Spectropolarimetric titrations with Zn^{2+}

Titrations were performed at 25°C under nitrogen atmosphere by adding aliquots of degassed zinc sulfate stock solution to a quartz cuvette (1 cm path length) containing peptide solution. Measurements were performed in 10 mM Tris-HCl buffer, pH 7.4 (I = 0.1 M from $NaClO_4$), 100–200 μM TCEP and a final peptide concentration of 5–50 μM, depending on the peptide length. The concentrations of the peptide solutions were determined with Ellman's reagent using a molar absorption coefficient of 14 150 M^{-1} cm^{-1}.[2] Spectra were measured on a Jasco J-1500 spectropolarimeter with a Peltier heating/cooling system. Three accumulations from 197 to 260 nm were averaged using a 5 nm band width, a 200 nm/min scanning speed, and a 1.0 nm data pitch. TCEP forms a very weak Zn^{2+} complex ($\log K_{ML}$ = 2.91) compared to the zinc hook peptides, and its metal-binding ability can be neglected.[3]

Potentiometric titrations

The protonation constants of the Hk4-Hk14 zinc hook peptides, depsipeptides, and stability constants of their Zn^{2+} complexes were determined at 25°C at 0.1 M ionic strength by potentiometric titration over a range of 2.5 to 10.8 (Molspin automatic titrator) under argon atmosphere using standardized 0.1 M NaOH as a titrant. The data were analyzed using SUPERQUAD software.[4]

Competitive titrations

The apparent formation constants of Zn^{2+} complexes with Hk23-45, Hk45VA, Hk45LA, and ZnHk45VALA were determined spectropolarimetrically (Jasco J-1500) at 25°C in the presence of HEDTA ($\log K$ = 12.2), EDTA ($\log K$ = 13.6) or TPEN ($\log K$ = 15.2).[5] In each experiment, 5 μM zinc hook peptide was incubated with 25 μM chelator and 0-25 μM $ZnSO_4$ in 10 mM Tris-HCl buffer (I = 0.1 M from $NaClO_4$) with 100 μM TCEP. Samples were incubated for 36 h under a nitrogen atmosphere. Formation of the $Zn(Hk)_2$ complex was monitored by measuring the ellipticity at 216 and 222 nm. The free Zn^{2+} concentration present in each sample after equilibration was calculated from the total chelator and metal concentrations, corrected for the Zn^{2+} transferred to the zinc hook peptide complex ($Zn(Hk)_2$). Calculations were performed using the Hyperquad Simulation and Speciation Software (HySS2009).[6] To obtain the apparent formation constants, first, we determined the normalized isotherms corresponding to complex formation by fitting with Hill's equation (equation (1)), where Θ, Θ_{min} and Θ_{max} are the observed minimum and maximum ellipticities, respectively. Here, n is the cooperativity index (Hill's coefficient), x is the free Zn^{2+} concentration at a

specific experimental point, and $[Zn^{2+}_{0.5}]$ is the free Zn^{2+} concentration at the half-point saturation of the $Zn(Hk)_2$ complex:

$$\Theta = \Theta_{\min}\left(\frac{x^n}{x^n+[Zn^{2+}{}_{0.5}]^n}\right) + \Theta_{max}\left(\frac{[Zn^{2+}{}_{0.5}]^n}{x^n+[Zn^{2+}{}_{0.5}]^n}\right) \qquad (1)$$

The obtained concentrations of free Zn^{2+}, referring to the half-point complex saturation $[Zn^{2+}_{0.5}]$, where half of the total peptide is in the form of $Zn(Hk)_2$ complex and half is in the metal-free form Hk, were subsequently used to calculate the apparent formation constants (K_{12}) based on equation (2), which was derived from equation (3):

$$K_{12} = \frac{1.25\ \mu M}{[Zn^{2+}{}_{0.5}]\times(5\ \mu M - 2\times 1.25\ \mu M)^2} \qquad (2)$$

$$K_{12} = \frac{[Zn(Hk)_2]}{[Zn^{2+}{}_{0.5}]\times[Hk]^2} \qquad (3)$$

Spectrophotometric determination of the pK_a values of cysteine thiols

The pK_a values of the cysteine thiols of Hk4-Hk45 were determined spectrophotometrically at 220 nm (Jasco J-760). The 100 µM peptide solutions in degassed 0.1 M $NaClO_4$ were titrated with concentrated NaOH in the pH range of 6–10.5 at 25°C. The dissociation constants (pK_{a1}^{SH}, pK_{a2}^{SH}) were obtained by data fitting to a two-binding-event equation, as described previously.[7] Similarly, the conditional dissociation constants of thiols in the presence of Zn^{2+} (pK_a') were determined by the pH-titration of 10 µM Hk4-Hk14 and Hk45 solutions with 4.95 µM Zn^{2+} in the pH range of 2.5–9. Absorbance values at 220 nm, corresponding to the formation of the ligand metal charge transfer (LMCT) bands of the complexes, were fitted to the logarithmic-form Hill's equation, as described elsewhere.[8]

Nuclear magnetic resonance (NMR) spectroscopy

NMR measurements of 5 mM $Zn(Hk6)_2$, $Zn(Hk10)_2$, $Zn(Hk12)_2$ and $Zn(Hk14)_2$ and metal-free Hk14 were performed in degassed 10% D_2O in H_2O (pH 7.4) on a DDR2 Agilent 600 MHz spectrometer equipped with a Penta probe.[9] The temperature coefficients of amide protons were derived from a series of 1D ^1H NMR spectra measured in the 5–35°C range. The exchange of amide protons with solvent was measured using the progressive saturation method.[10] Eight data points with presaturation times in the range of 0.1−8 s were collected. The sum of interscan delay and presaturation time was kept constant at 8 s. The exchange (k_{ex}) and longitudinal (ρ) relaxation rates of the amide protons were fit to the amide proton

signal intensities.[11] Amide proton accessibility to solvent was described in the framework of protection factors.[12] The 2D homonuclear total correlation spectroscopy (TOCSY) (mixing time 80 ms)[13], rotating-frame nuclear Overhauser effect (ROESY) (mixing time 300 ms)[14] and heteronuclear $^{1}H/^{15}N$ and $^{1}H/^{13}C$ heteronuclear single quantum coherence (HSQC) (^{13}C HSQC was recorded with the offset, spectral widths, and $^{13}C–^{1}H$ coupling constant adjusted to aliphatic carbons)[15] spectra recorded at 25°C were used to obtain assignments of the ^{1}H, ^{13}C and ^{15}N resonances (Table S10). Time-domain data were acquired using States-TPPI quadrature detection.[16] All chemical shifts in ^{1}H NMR spectra are reported with respect to external bis(succinimidyl)-2,2,7,7-suberate-d4 (DSS-d4). The chemical shifts of the ^{13}C and ^{15}N signals were referenced indirectly using the 0.251449530 and 0.101329118 frequency ratios for $^{13}C/^{1}H$ and $^{15}N/^{1}H$, respectively.[17] Zero filling and a 90°-shifted squared sine-bell filter were performed prior to Fourier transformation. Processed spectra were analyzed with SPARKY software.[18]

Structural calculations

The standard approach to structural calculations for a zinc hook complex with the Hk14 peptide based on automatic assignment of interproton distances derived from the 2D ROESY spectrum failed because of the ambiguity of intrasubunit and intersubunit signals and the high symmetry of the homodimer complex.[19] Therefore, the available Rad50 protein crystallographic structure (PDB ID: 1L8D) was investigated as a model in our structural analysis.[20] A list of possible through-space correlations was prepared for a corresponding fragment (protons were added to the respective structure fragment with WHATIF).[21] Pairs of closely spaced protons ($d \leq 5$ Å) were used to create the list of distances for comparison with the list of peaks obtained from the ROESY spectrum.

Hydrogen-deuterium exchange mass spectrometry (HDX MS)

The experiments were conducted in the exchange mode, e.g., hydrogen into deuterium. The spectra for each zinc hook peptide were measured under four conditions: peptide in H_2O, peptide- Zn^{2+} complex in H_2O, peptide in D_2O and peptide-Zn^{2+} complex in D_2O. The samples measured by ion trap were prepared by mixing the peptide and $ZnCl_2$ at a 5:1 molar ratio at a peptide concentration of ~ 50 nM in 10-mM $(NH_4)_2CO_3$ pH 7.4 in D_2O or H_2O in a final volume of 200 μl. Mass spectra were measured on an amaZon SL ion trap spectrometer (Bruker, Germany) in electron-transfer dissociation (ETD) fragmentation mode using a

capillary voltage of 4 000 V, an end plate offset of -500 V, a flow rate of 5.00 μl/min, a nebulizer pressure of 10.0 psi, and a dry argon flow rate of 3.0 l/min at 150°C in positive ion mode. The ETD fragmentation spectra of the ions of interest were recorded with the amplitude refined for each parent ion. The collision-induced dissociation (CID) spectra of the Hk14 complex were also collected using a Maxis Impact qTOF spectrometer (Bruker, Germany) with standard settings in positive ion mode (capillary voltage 2 000 V, end plate offset -500 V, nebulizer 0.5 bar, dry argon 4.0 l/min, 180°C). The collision energy was adjusted for each parent ion to obtain a good fragmentation spectrum. Samples measured by quadrupole time of flight (qTOF) were diluted directly before measurement by a factor of 4,000 to prevent detector saturation. The spectra recorded for peptides and their complexes in H_2O were treated as the reference and control in terms of fragmentation. For each deuterated sample, isotopic profiles of 1:1 and 1:2 metal-to-peptide complexes were compared with the theoretical isotopic profile of fully deuterated species, and the signals corresponding to complexes with various numbers of protected protons were analyzed. Subsequently, fragmentation ions were analyzed in the same manner. Comparison of the number of protected protons in each fragmentation peak and the peptide sequence provided information regarding possible hydrogen bond donor locations.

Isothermal titration calorimetry (ITC)

The binding of Zn^{2+} to Hk peptides was monitored using a nano-ITC calorimeter (TA Waters, USA) at 25°C with a cell volume of 1 ml. All experiments were performed in HEPES buffer (I = 0.1 M from NaCl) at pH 7.4 under an argon atmosphere. The Hk peptide (titrant) concentration was 1.3 mM, whereas the metal (titrate) concentration was 50 μM. After temperature equilibration, successive injections of the titrant were made into the reaction cell with 6.82 μl increments at 400 s intervals with stirring at 200 rpm. Control experiments to determine the heats of titrant dilution were performed using identical injections in the absence of Zn^{2+}. The net reaction heat was obtained by subtracting the heat of dilution from the corresponding total heat of reaction. The titration data were analyzed using NanoAnalyze (version 3.3.0) and Origin software (version 8.1) and were fitted to a sequential binding model to account for the formation of ZnHk and $Zn(Hk)_2$ during the course of titration.

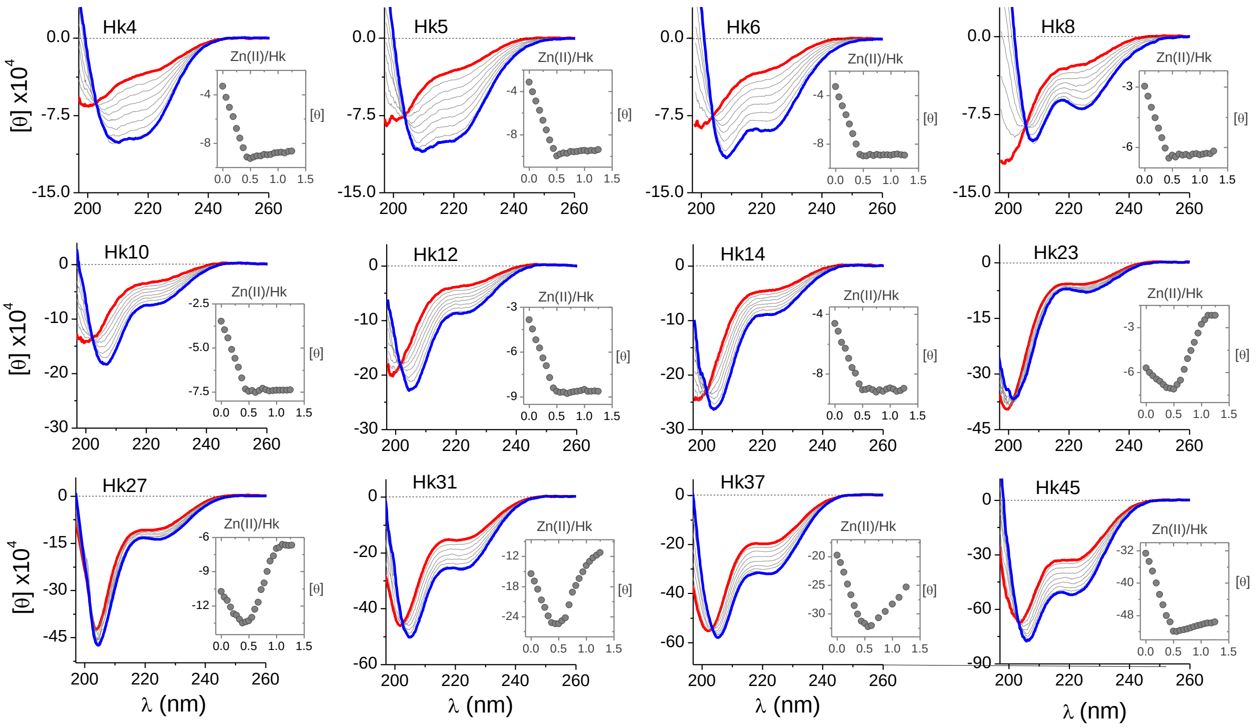

Figure S1. Spectropolarimetric titrations of zinc hook peptides with Zn^{2+} in 5 mM Tris buffer at pH 7.4, 25°C, *I* = 0.1 M (from $NaClO_4$) with 100 μM TCEP. Red and blue indicate free peptide and a Zn^{2+}-to-peptide molar ratio of 0.5:1 ($Zn(Hk)_2$ complex), respectively. Spectra collected at higher ratios are omitted for clarity. Insets present the dependence of the ellipticity on the reactant molar ratio, as monitored at 222 nm.

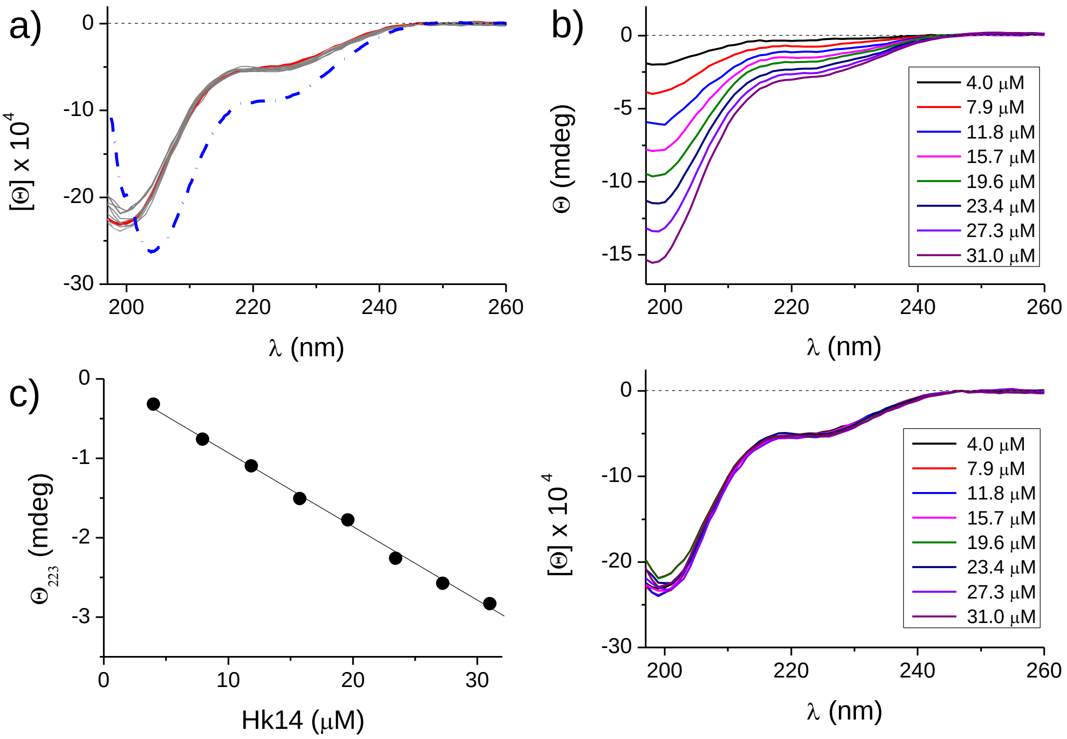

Figure S2. CD titrations of Hk14 in the absence of Zn^{2+} ions; a) Spectra of 15 μM Hk14 titrated with HEPES buffer, pH 7.4; b) Spectra of Hk14 peptide recorded at different concentrations; c) Dependence of ellipticity of Hk14 as a function of peptide concentration; d) CD spectra of Hk14 recorded at different concentrations (see graph c) converted to molar ellipticity [Θ] (cm^2 $dmol^{-1}$).

Table S1. Cumulative protonation and Zn^{2+} stability constants (log β_{ijk})[a] of zinc hook peptide complexes determined potentiometrically at 25°C, I = 0.1 M (from KNO_3).[b] *n.d.* denotes not determined under the used conditions.

log β_{ijk}	Zinc hook peptide						
	Hk4	Hk5	Hk6	Hk8	Hk10	Hk12	Hk14
HL	9.362(9)	9.15(1)	8.98(1)	10.63(2)	10.64(2)	10.71(3)	10.74(3)
H_2L	17.54(1)	17.19(1)	16.79(1)	19.51(2)	19.53(3)	20.88(3)	21.27(1)
H_3L	-	-	-	27.25(3)	26.89(3)	29.69(3)	30.26(2)
H_4L	-	-	-	31.50(2)	31.08(4)	37.07(4)	37.65(2)
H_5L	-	-	-	-	-	41.27(7)	42.14(3)
H_6L	-	-	-	-	-	-	45.69(3)
ZnH_2L	-	-	-	-	-	31.91(6)	32.51(4)
ZnHL	-	-	-	20.8(1)	21.70(5)	-	-
ZnL	10.17(5)	9.63(8)	9.83(8)	-	-	-	-
ZnH_4L_2	-	-	-	-	-	64.18(4)	65.52(5)
ZnH_3L_2	-	-	-	-	-	55.61(8)	56.25(7)
ZnH_2L_2	-	-	-	43.02(4)	43.47(3)	45.38(9)	45.75(6)
$ZnHL_2$	-	-	-	33.39(8)	33.97(7)	34.3(1)	*n.d.*
ZnL_2	20.55(3)	20.33(3)	20.71(1)	*n.d.*	*n.d.*	*n.d.*	*n.d.*

[a] $\beta M_iH_jL_k = [M_iH_jL_k]/([M]^i[H]^j[L]^k)$, where [L] is the concentration of fully deprotonated zinc hook peptide.
[b] Standard deviations are given as provided by SUPERQUAD calculations and refer to the last digit.(1)

Procedure of the calculation of formation constant (log K_{12}) for $Zn(Hk14)_2$ based on potentiometric protonation and stability constants derived from potentiometric titrations presented in Table S1:

a) Use any simulation and speciation software (e.g. Hyperquad) to calculate concentration values of $[Zn^{2+}]$ (free Zn^{2+}), $[Zn(Hk)_2]$ (complex in equilibrium) and [Hk] (free ligand). For that purpose prepare input file with all protonation constants and stability constants listed for any hook peptide listed in Table S1. Define total concentrations of the reagents, for example 5 μM of the peptide and 1.25 μM of total Zn^{2+}. Define pH range or pH of interest for which speciation should be calculated. Run the calculations.

b) Use calculated values of $[Zn^{2+}]$, $[Zn(Hk)_2]$ and [Hk] concentrations at pH of interest (e.g. 7.4) to calculate the formation constant for that pH according to the following equation:

$$K_{12} = \frac{[\mathrm{Zn(Hk)_2}]}{[\mathrm{Zn^{2+}}][\mathrm{Hk}]^2}$$

In the case of Hk14 and pH 7.4 one obtains: $[Zn^{2+}] = 1.28\times10^{-14}$ M, $[Zn(Hk14)_2] = 1.25\times10^{-6}$ M and $[Hk14] = 2.5\times10^{-6}$ M and $K_{12} = 1.56\times10^{19}$. Logarithmic value of K_{12}, $\log K_{12} = 19.19$. The value corresponds to that listed in Table 1 in the main manuscript.

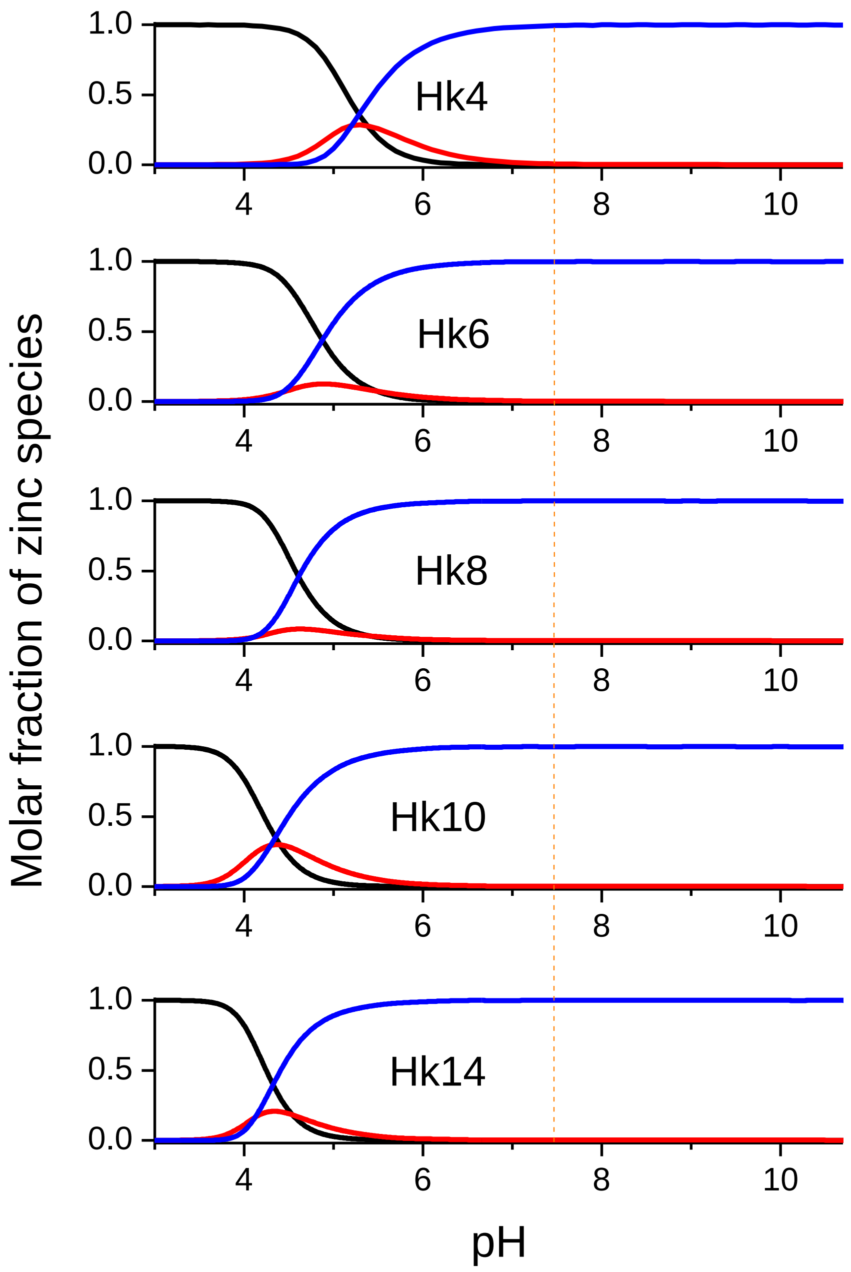

Figure S3. Speciation plots of selected zinc hook peptides for different reactant concentrations used in the potentiometric studies: 1 mM hook peptide and 0.5 mM Zn^{2+}. Black, red and blue lines represent unbound Zn^{2+}, monomeric (ZnHk) and dimeric ($Zn(Hk)_2$) fractions, respectively. The orange line corresponds to pH 7.4.

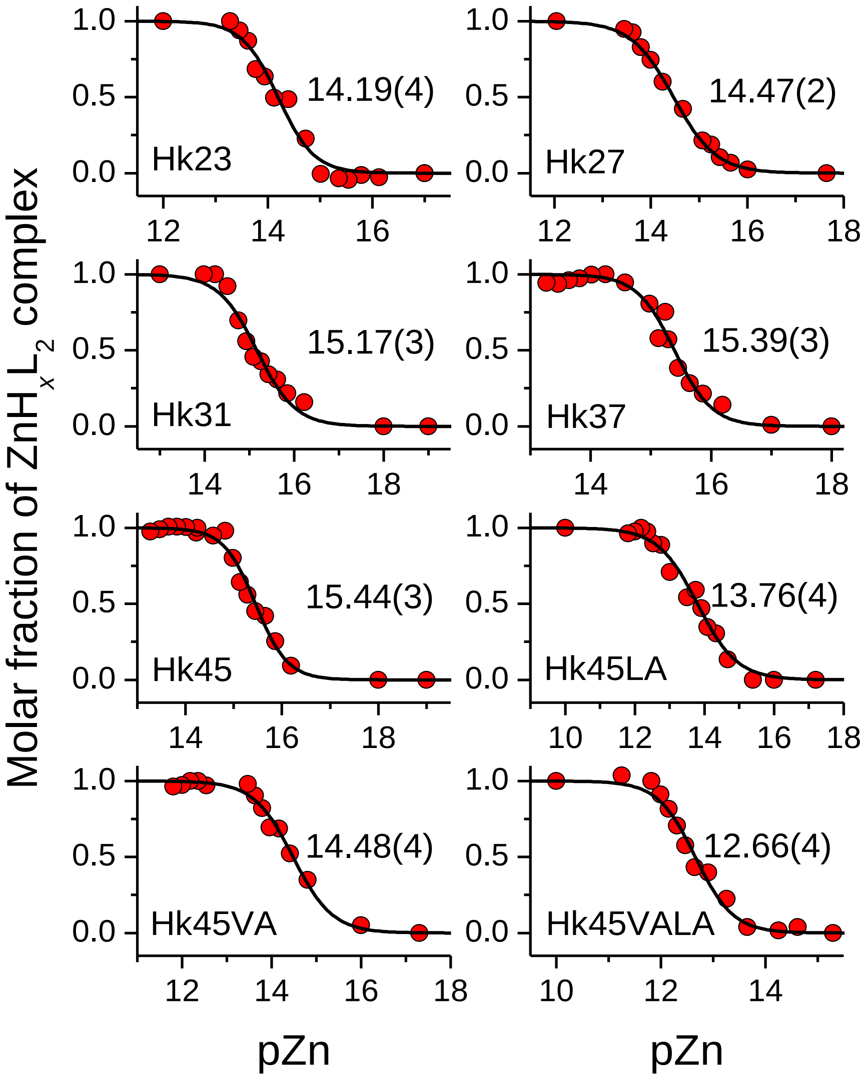

Figure S4. Isotherms of Zn^{2+} binding to Hk23, Hk27, Hk31, Hk37, Hk45, Hk45LA, Hk45VA, and Hk45LAVA zinc hook peptides in the presence of zinc chelators.

Table S2. ^{1}H chemical shifts of Zn^{2+}- complexes of zinc hook peptides in ppm.

Zinc hook peptide	Atom name	Hk14	Hk10	Hk8	Hk6
acetyl group	H_M	1.990	2.02	2.009	1.854
Ala440	H_N<br>H_α<br>H_β	8,265<br>4.248<br>1.338	not applicable	not applicable	not applicable
Lys441	H_N<br>H_α<br>H_β<br>H_γ<br>H_δ<br>H_ε	8.386<br>4.343<br>1.731; 1.854<br>1.464; 1.402<br>1.639<br>2.968	not applicable	not applicable	not applicable
Gly442	H_N<br>H_α	8.392<br>3.986	8.347<br>4.011; 3.898	not applicable	not applicable
Lys443	H_N<br>H_α<br>H_β<br>H_γ<br>H_δ<br>H_ε	8.156<br>4.577<br>1.465; 1.395<br>1.279; 1.147<br>1.586<br>2.956	8.200<br>4.576<br>1.469; 1.396<br>1.283; 1.155<br>1.583<br>2.958	8.197<br>4.206<br>1.492<br>1.212; 1.150<br>1.607<br>2.959	not applicable
Cys444	H_N<br>H_α<br>H_β	8.634<br>4.572<br>2.655; 3.257	8.667<br>4.559<br>2.657; 3.252	8.424<br>4.689<br>2.802; 3.315	8.197<br>4.667<br>3.251; 2.774
Pro445	H_α<br>H_β<br>H_γ<br>H_δ	4.429<br>2.360; 2.060<br>2.156; 1.989<br>3.930; 4.312	4.424<br>2.376; 2.050<br>2.144; 1.981<br>3.922; 4.303	4.431<br>2.360; 2.041<br>2.118; 1.988<br>3.944; 4.237	4.432<br>2.356; 2.018<br>2.103; 2.007<br>4.205; 3.915
Val446	H_N<br>H_α<br>H_β<br>$H_{\gamma1}$<br>$H_{\gamma2}$	8.704<br>4.116<br>1.997<br>0.966<br>0.944	8.709<br>4.1091<br>1.986<br>0.964<br>0.934	8.874<br>4.056<br>1.941<br>0.958<br>0.960	8.889<br>4.030<br>1.989<br>0.954<br>0.958
Cys447	H_N<br>H_α<br>H_β	8.216<br>4.916<br>2.903; 3.235	8.235<br>4.898<br>2.901; 3.215	8.308<br>4.923<br>2.944; 3.139	8.285<br>4.902<br>3.137; 2.921
Gly448	H_N<br>H_α	7.712<br>3.865; 4.114	7.723<br>3.854; 4.109	7.823<br>3.862; 4.112	7.977<br>4.078; 3.917
Arg449	H_N<br>H_α<br>H_β<br>H_γ<br>H_δ	7.909<br>4.401<br>1.781; 1.908<br>1.769<br>3.177; 3.260	7.962<br>4.374<br>1.785; 1.918<br>1.737<br>3.103; 3.252	7.962<br>4.374<br>1.785; 1.918<br>1.737<br>3.103; 3.252	8.403<br>4.339<br>1.886; 1.775<br>1.759; 1.613<br>3.152
Glu450	H_N<br>H_α<br>H_β<br>H_γ	not assigned<br>4.238<br>1.974<br>2.296	not assigned<br>4.224<br>1.971; 2.314<br>2.314	8.621<br>4.358<br>1.964; 1.883<br>2.179	not applicable
Leu451	H_N<br>H_α<br>H_β	8.473<br>4.346<br>1.481; 1.545<br>1.460	8.529<br>4.234<br>1.471; 1.550<br>1.479	not applicable	not applicable

	H_γ<br>$H_{\delta 1}$<br>$H_{\delta 2}$	0.783<br>0.863	0.766<br>0.840		
Thr452	H_N<br>H_α<br>H_β<br>$H_{\gamma 2}$	8.265<br>4.346<br>4.247<br>1.181	not applicable		
Asp453	H_N<br>H_α<br>H_β	8.322<br>4.597<br>2.635; 2.665			
NH_2 group	H_1<br>H_2	7.488<br>7.053	7.777<br>7.113	7.666<br>6.965	7.750<br>7.150

Table S3. ^{13}C and ^{15}N chemical shifts of Zn^{2+} complexes of zinc hook peptides in ppm.

Zinc hook peptide	atom name	Hk14	Hk10	Hk8	Hk6
acetyl group	C_M	24.47	24.58	24.44	24.06
Ala440	N_H<br>C_α<br>C_β	120.16<br>56.14<br>19.32	not applicable	not applicable	not applicable
Lys441	N_H<br>C_α<br>C_β<br>C_γ<br>C_δ<br>C_ε	120.16<br>56.14<br>33.34<br>35.29<br>28.95<br>42.12			
Gly442	N_H<br>C_α	109.15<br>45.03	114.33<br>45.08		
Lys443	N_H<br>C_α<br>C_β<br>C_γ<br>C_δ<br>C_ε	118.99<br>not assigned<br>not assigned<br>24.94<br>29.25<br>42.15	119.12<br>not assigned<br>35.21<br>24.90<br>29.14<br>42.11	126.88<br>55.93<br>33.77<br>25.04<br>29.44<br>42.06	
Cys444	N_H<br>C_α<br>C_β	127.09<br>not assigned<br>31.4	127.48<br>not assigned<br>31.44	129.28<br>not assigned<br>31.93	130.977<br>not assigned<br>32.099
Pro445	C_α<br>C_β<br>C_γ<br>C_δ	64.33<br>32.53<br>27.19<br>51.81	64.29<br>32.54<br>27.18<br>51.74	64.16<br>32.42<br>27.22<br>51.70	64.41<br>32.19<br>27.25<br>51.46
Val446	N_H<br>C_α<br>C_β<br>$C_{\gamma1}$<br>$C_{\gamma2}$	121.60<br>65.18<br>32.95<br>21.73<br>23.43	121.61<br>65.14<br>32.96<br>21.77<br>23.43	122.03<br>65.38<br>33.00<br>21.58<br>22.92	122.17<br>65.32<br>not assigned<br>21.76<br>22.89
Cys447	N_H<br>C_α<br>C_β	118.43<br>not assigned<br>31.42	118,.<br>not assigned<br>31.60	118.51<br>not assigned<br>31.96	118.845<br>not assigned<br>31.96
Gly448	N_H<br>C_α	111.58<br>46.58	111.58<br>46.50	112.24<br>46.41	112.39<br>46.15
Arg449	N_H<br>C_α<br>C_β<br>C_γ<br>C_δ	120.57<br>56.25<br>31.63<br>27.73<br>43.54	120.80<br>56.45<br>31.56<br>27.69<br>43.46	121.36<br>57.05<br>31.19<br>29.09<br>43.53	122.07<br>56.33<br>31.13<br>27.86<br>43.61
Glu450	N_H<br>C_α<br>C_β<br>C_γ	not assigned<br>not assigned<br>30.10<br>36.59	not assigned<br>57.61<br>29.83<br>36.41	121.86<br>55.74<br>31.63<br>36.38	not applicable
Leu451	N_H<br>C_α<br>C_β	126.09<br>55.23<br>42.25<br>27.34	126.58<br>55.32<br>42.64<br>27.41	not<br>applicab<br>le	

	C_{γ}<br>$C_{\delta1}$<br>$C_{\delta2}$	25.41<br>23.22	25.19<br>23.10		
Thr452	N_H<br>C_{α}<br>C_{β}<br>$C_{\gamma2}$	129.94<br>61.71<br>69.92<br>21.51	not applicable		
Asp453	N_H<br>C_{α}<br>C_{β}	122.85<br>not assigned<br>41.51			
NH_2 group	N	107.39	107.22	108.39	108.2

Table S4. Experimentally determined values of exchange rates (k_{ex}) and longitudinal relaxation rates (ρ) of amide protons (H_N) for Zn^{2+} complexes with Hk6-Hk10 and Hk14 zinc hook peptides and simulated values for Hk14, assuming random-coil conformation at pH 7.4 and 25°C. *n.a.* and *n.d.* denote not applicable and not determined, respectively.

Proton amide	Hk6		Hk8		Hk10		Hk14		Hk14 random coil
	k_{ex} (s^{-1})	ρ (s^{-1})	k_{ex} (s^{-1})	ρ (s^{-1})	k_{ex} (s^{-1})	ρ (s^{-1})	k_{ex} (s^{-1})	ρ (s^{-1})	k_{ex} (s^{-1})
Ala440	*n.a.*	*n.a.*	*n.a.*	*n.a.*	*n.a.*	*n.a.*	0.211(4)[a]	0.82(2)[a]	*n.a.*[e]
Lys441	*n.a.*	*n.a.*	*n.a.*	*n.a.*	*n.a.*	*n.a.*	14(2)[b]	3.2(2)[b]	40.74
Gly442	*n.a.*	*n.a.*	*n.a.*	*n.a.*	16.3(3)	1.16(2)	14(2)[b]	3.2(2)[b]	109.64
Lys443	*n.a.*	*n.a.*	2.54(2)	1.57(1)	10.8(3)	2.15(2)	6.4(2)	0.90(3)	60.25
Cys444	3.63(1)	1.736(3)	0.81(2)	1.71(3)	0.608(5)	1.46(1)	0.65(1)	1.18(2)	245.46
Pro445	*n.a.*	*n.a.*	*n.a.*	*n.a.*	*n.a.*	*n.a.*	*n.a.*	*n.a.*	*n.a.*
Val446	0.143(3)	1.36(3)	0.201(5)	1.38(3)	0.047(5)	0.77(7)	0.146(5)	0.80(2)	5.13
Cys447	0.89(2)	1.82(4)	0.37(2)	1.43(5)	0.084(2)	0.91(2)	0.211(4)[a]	0.82(2)[a]	134.89
Gly448	4.93(4)	1.883(3)	0.443(5)	1.43(1)	0.108(6)	0.87(5)	0.266(7)	0.81(2)	295.11
Arg449	11.2(4)	1.93(2)	1.07(2)	3.6(3)	0.355(3)	1.30(1)	0.34(2)	0.91(4)	79.43
Glu450	*n.a.*	*n.a.*	8(2)	*n.d.*[c]	*n.d.*[c]	*n.d.*[c]	*n.d.*[c]	*n.d.*[c]	22.91
Leu451	*n.a.*	*n.a.*	*n.a.*	*n.a.*	1.16(3)	1.86 ± 0.03	0.94(3)	1.60(4)	8.32
Thr452	*n.a.*	*n.a.*	*n.a.*	*n.a.*	*n.a.*	*n.a.*	0.211(4)[a]	0.82(2)[a]	23.44
Asp453	*n.a.*	*n.a.*	*n.a.*	*n.a.*	*n.a.*	*n.a.*	13.2(2)	1.47(1)	35.48

[a] Three amide protons of $Zn(Hk14)_2$ with undistinguishable proton chemical shifts. [b] Two amide protons of $Zn(Hk14)_2$ with undistinguishable proton chemical shifts. [c] The amide signal of Glu450 was too broadened to measure its k_{ex} and ρ. [d] Simulated exchange rates of the amide protons of Hk14 with solvent, calculated for random coil peptides assuming 25ºC and pH 7.4. [e] Calculation model for exchange rates considering only the preceding residue.

Table S5. Values of protection factors and temperature coefficients calculated for Zn^{2+} complexes with Hk6, Hk8, Hk10 and Hk12 zinc hook peptides at pH 7.4 and 25°C. *n.a.* and *n.d.* denote not applicable and not determined, respectively.

Proton amide	Hk6		Hk8		Hk10		Hk14	
	Protection factor	$-\Delta\delta/\Delta T$ (ppb)	Protection factor	$-\Delta\delta/\Delta T$ (ppb)	Protection factor	$-\Delta\delta/\Delta T$ (ppb)	Protection factor	$-\Delta\delta/\Delta T$ (ppb)
Ala440	*n.a.*	*n.a.*	*n.a.*	*n.a.*	*n.a.*	*n.a.*	*n.d.*[b]	9.4
Lys441	*n.a.*	*n.a.*	*n.a.*	*n.a.*	*n.a.*	*n.a.*	*n.d.*[b]	8.6
Gly442	*n.a.*	*n.a.*	*n.a.*	*n.a.*	*n.d.*[a]	8.7	*n.d.*[b]	8.6
Lys443	*n.a.*	*n.a.*	*n.d.*[a]	9.8	6.00	9.3	9.40	8.4
Cys444	*n.d.*[a]	7.2	301.55	5.5	403.85	2.1	376.88	1.6
Pro445	*n.a.*	*n.a.*	*n.a.*	*n.a.*	*n.a.*	*n.a.*	*n.a.*	*n.a.*
Val446	35.87	1.4	25.57	2.7	108.46	2.4	35.11	2.1
Cys447	151.39	0.4	365.55	1	1605.83	1.5	*n.d.*[b]	1.3
Gly448	59.91	3.3	665.56	3.4	2737.57	2.2	1109.44	2.2
Arg449	7.12	5.8	74.30	4.9	223.62	3.8	230.90	3.2
Glu450	*n.a.*	*n.a.*	3.01	6.8	*n.d.*[b]	*n.d.*[b]	*n.d.*[b]	*n.d.*[b]
Leu451	*n.a.*	*n.a.*	*n.a.*	*n.a.*	7.20	7.2	8.88	5.8
Thr452	*n.a.*	*n.a.*	*n.a.*	*n.a.*	*n.a.*	*n.a.*	*n.d.*[b]	9.4
Asp453	*n.a.*	*n.a.*	*n.a.*	*n.a.*	*n.a.*	*n.a.*	2.68	*n.d.*[b]

[a] Calculation model considering only the preceding residue.
[b] Not determined because of signals overlapping or a lack of clear assignments (see footnote of Table S4).

a)

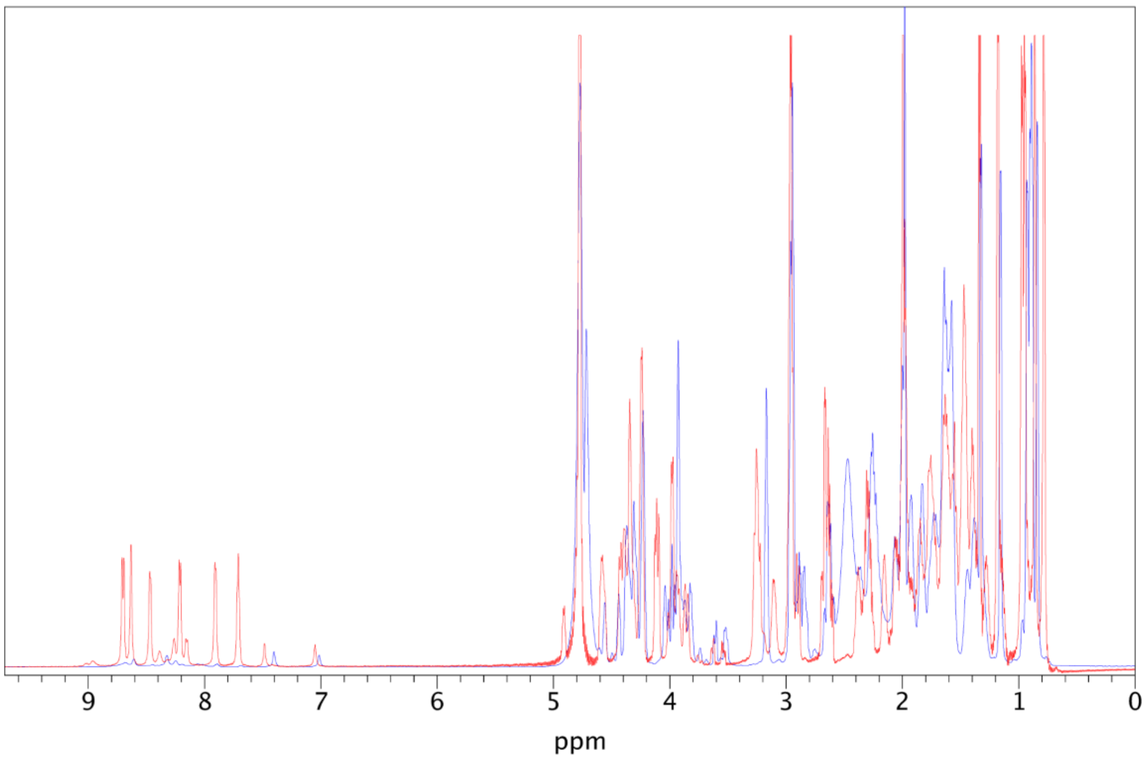

b)

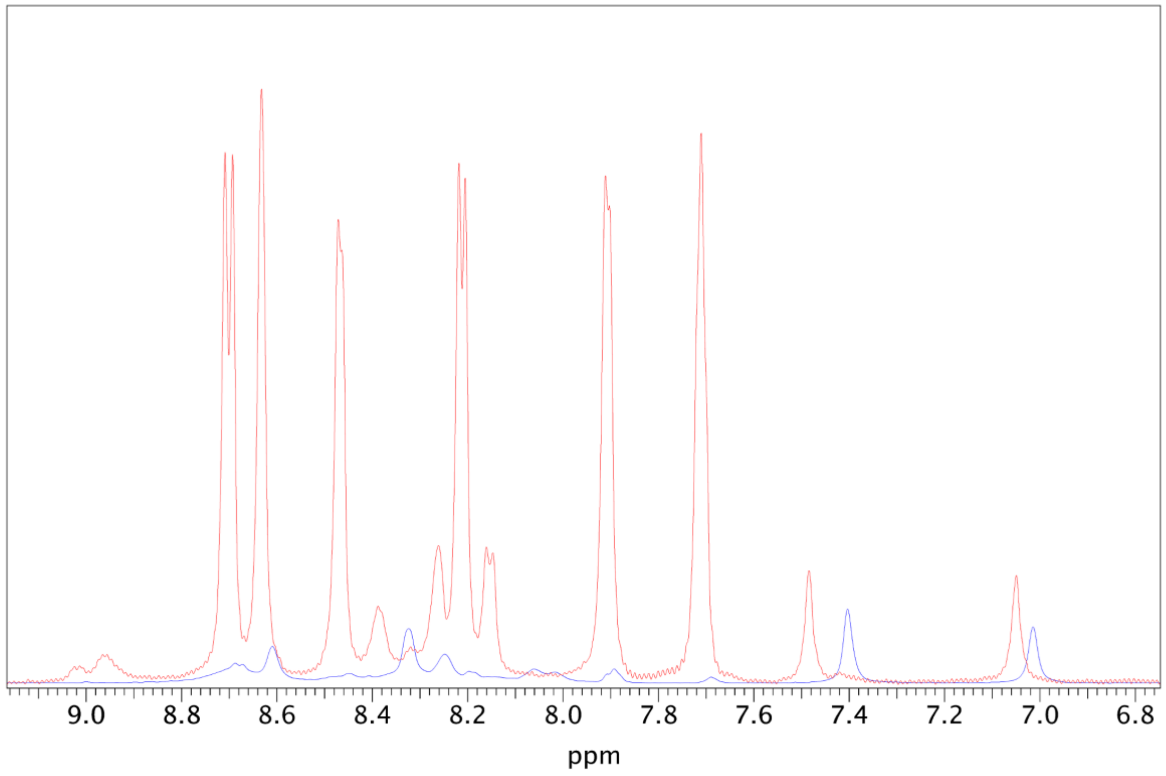

Figure S5. Overlaid 1D ^1H NMR spectra of free (blue) and Zn^{2+} complexed (red) Hk14 peptides (spectra were determined with 1 s of saturation of the H_2O signal). a) Full chemical shift range; b) amide region of 1D ^1H NMR spectra.

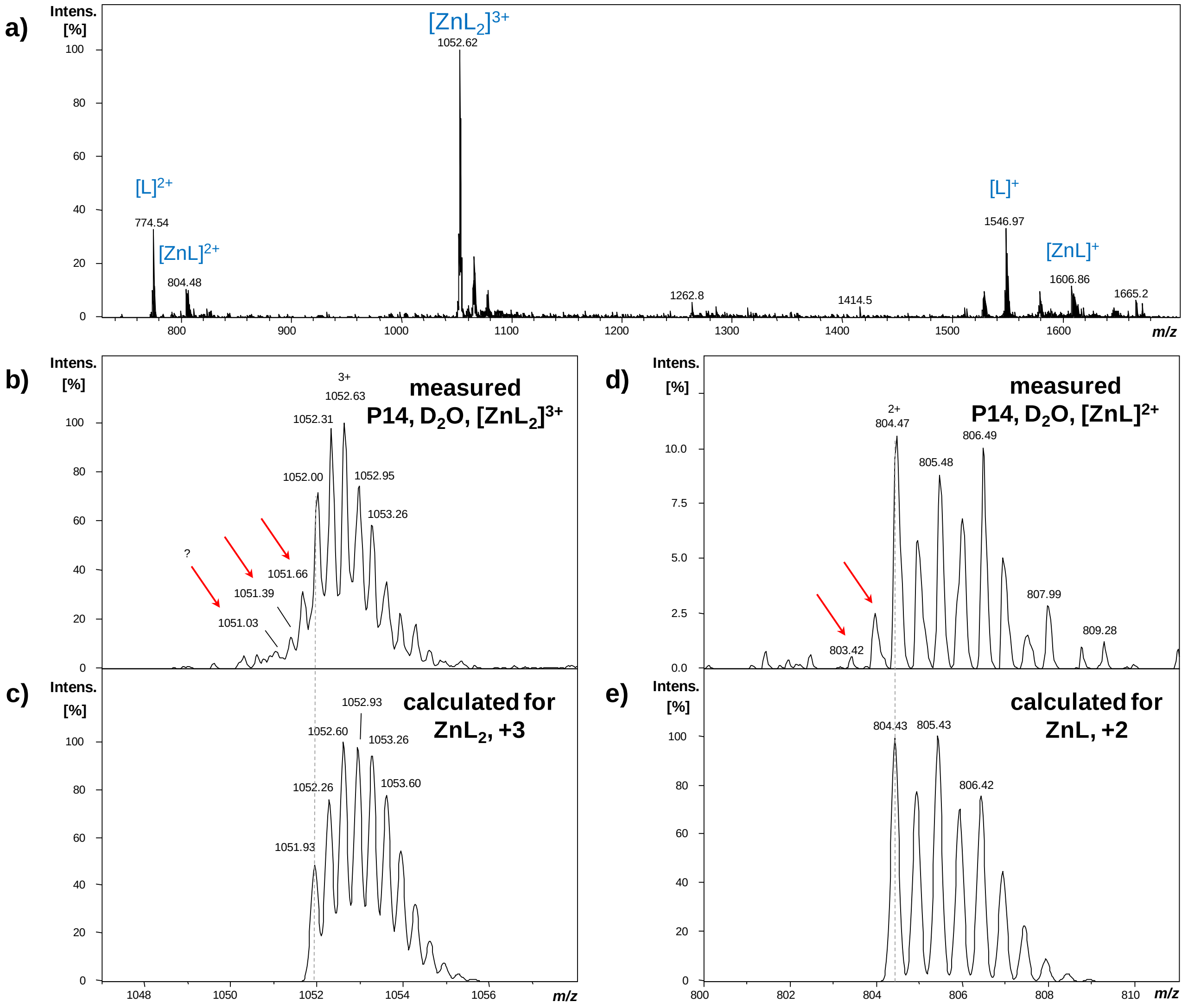

Figure S6. Example of HDX MS analysis: mass spectrum of Hk14 in D_2O measured on an AmaZonSL (ion trap) instrument: a) full spectrum; b) measured isotopic profile of complex $Zn(Hk14)_2$ ($[ZnL_2]^{3+}$); c) simulated isotopic profile for $C_{124}H_{160}D_{57}N_{40}O_{40}S_4Zn$, +3; d) measured isotopic profile of complex ($[ZnL]^{2+}$); and e) simulated isotopic profile for $C_{62}H_{80}D_{28}N_{20}O_{20}S_2Zn$, +2. Arrows indicate possible protected protons. Dashed grey lines indicate monoisotopic peaks. Simulations were conducted using DataAnalysis 4.0 (Bruker Daltonics software).

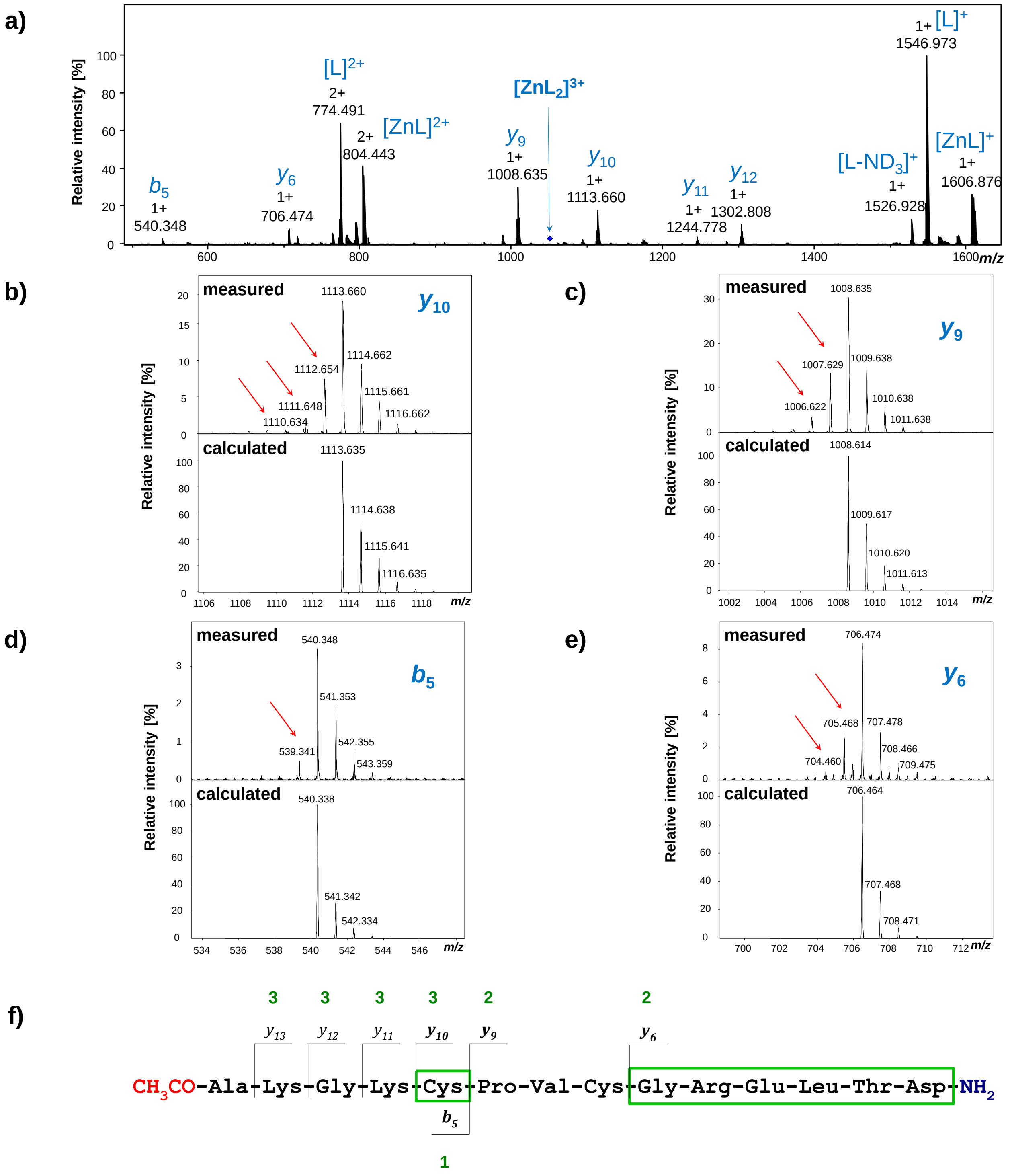

Figure S7. Example of HDX MS analysis: fragmentation mass spectrum of deuterated Zn(Hk14)$_2$ complex ([ZnL$_2$]$^{3+}$) (parent ion 1052.0 *m/z*, isolation with 5.0 *m/z* and collision energy 50 eV). a) Whole spectrum with annotated fragment ions; b)–e) isotopic envelopes for selected ion peaks: measured (top) and calculated (fully deuterated, bottom). Red arrows indicate protected protons. From fragments y_{10} (three protected protons) and y_9 (two protected protons), one protected proton on the Cys444 amide can be assigned, as confirmed by fragment b_5 (one protected proton). Fragment y_6 indicates two protected protons in the Gly-Arg-Glu-Leu-Thr-Asp region. The green box shows the localization of the protected protons. Simulations were conducted using DataAnalysis 4.0 (Bruker Daltonics software).

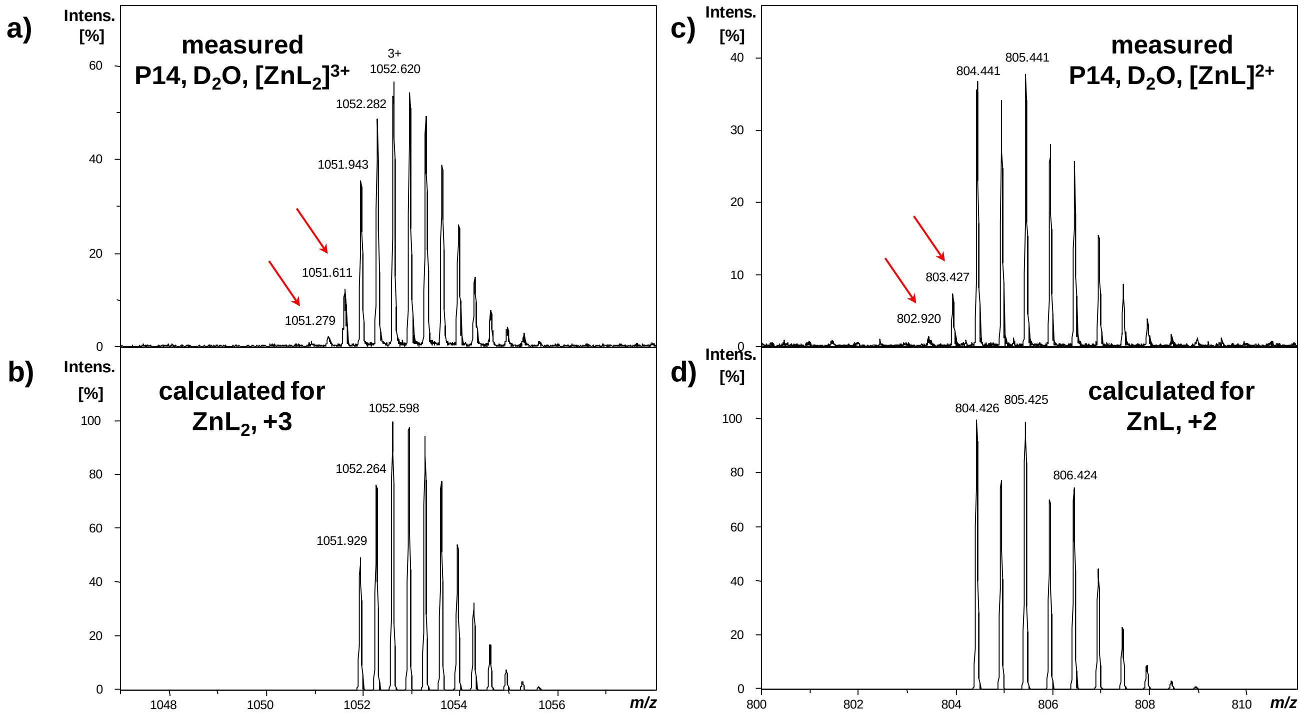

Figure S8. Example of HDX MS analysis: comparison of measured (upper panels) and calculated (lower panels) isotopic profiles of Hk14 Zn^{2+} complexes in D_2O measured on Maxis Impact (qTOF): **a)** measured isotopic profile of complex $Zn(Hk14)_2$ ($[ZnL_2]^{3+}$); b) Simulated isotopic profile for $C_{124}H_{160}D_{57}N_{40}O_{40}S_4Zn$, +3; c) Measured isotopic profile of complex Zn(Hk14) $[ZnL]^{2+}$; d) Simulated isotopic profile for $C_{62}H_{80}D_{28}N_{20}O_{20}S_2Zn$, +2. Arrows indicate possible protected protons. Simulations were conducted with DataAnalysis 4.0 (Bruker Daltonics software). The same number of protected protons deduced from spectra measured on ion trap and qTOF points out the importance of the molecule size (which makes other protected protons undetectable).

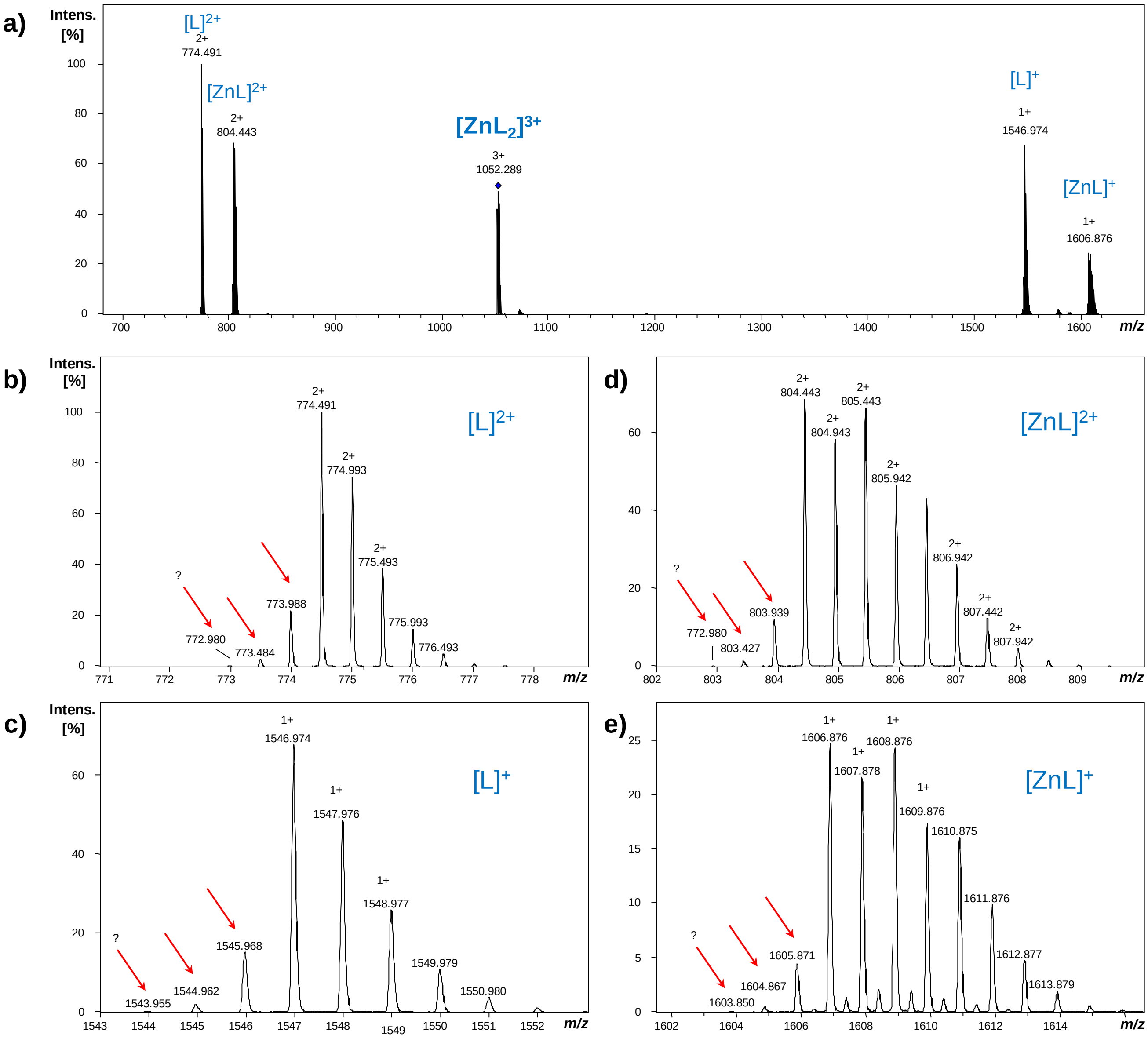

Figure S9. Example of HDX MS analysis: fragmentation mass spectrum of deuterated Zn(Hk14)$_2$ ([ZnL$_2$]$^{3+}$) complex in D$_2$O, measured on Maxis Impact (CID). Parent ion 1052.0 *m/z*, isolation width 5.0 *m/z*, collision energy 10 eV. a) Full fragmentation spectrum shows dissociation of a complex ZnL$_2$ into subunits: ZnL and L (charge states were omitted for clarity). b)–d) Isotopic envelopes of selected daughter ions. Red arrows indicate protected protons.

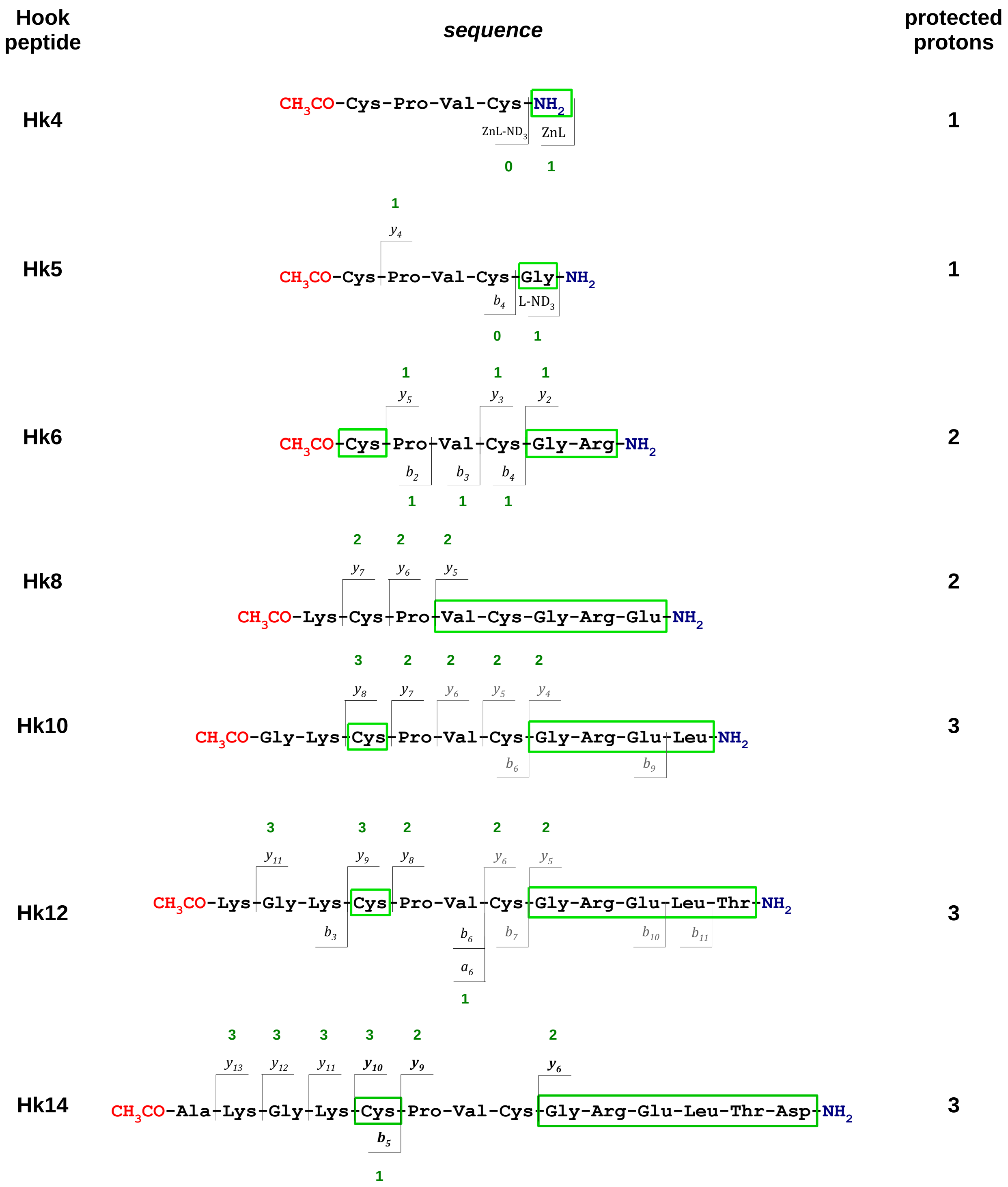

Figure S10. Localization of protected protons based on ESI-MS spectra. Green boxes indicate regions with protected amide protons. Single-residue resolution was obtained in only a few cases. The value above each fragment denotes the number of protected protons in that fragment. The right column presents the total number of protected protons in each peptide determined by this method.

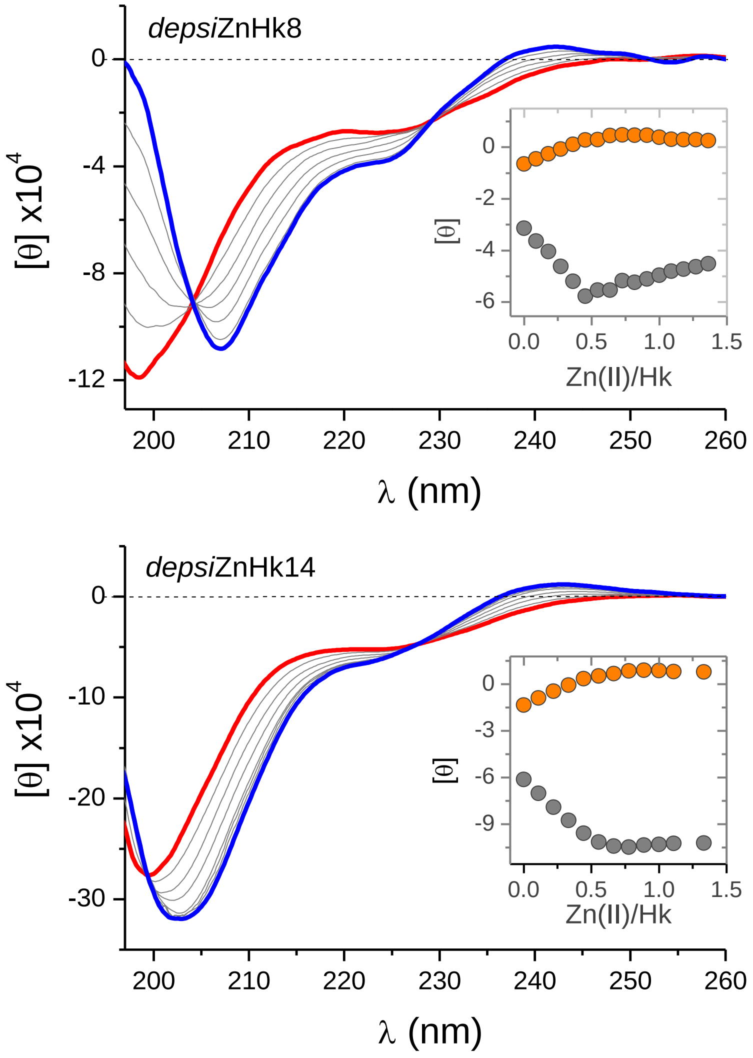

Figure S11. Spectropolarimetric titration of zinc hook depsipeptides with Zn^{2+} in 5 mM Tris buffer (I = 0.1 M from $NaClO_4$) at pH 7.4 and 25°C. Red and blue correspond to free peptide and a Zn^{2+}-to-peptide ratio of 0.5:1 ($Zn(Hk)_2$ complex), respectively. Spectra collected at higher ratios were omitted for clarity. Insets present the dependence of the molar ellipticity of the zinc hook peptide, as monitored at 222 (grey circles) and 242 nm (orange circles), on the Zn^{2+}-to-Hk peptide molar ratio.

Table S6. Cumulative protonation and Zn^{2+} stability constants (log β_{ijk})[a] of zinc hook peptide complexes determined potentiometrically at 25°C, I = 0.1 M (from KNO_3).[b] *n.d.* denotes not determined under the used conditions.

log β_{ijk}	Zinc hook peptide						
	Hk4VA	Hk4PA	Hk4PAVA	*depsi*Hk8	*depsi*Hk14	*depsi*Hk14LA	*depsi*Hk14VALA
HL	9.265(8)	9.232(9)	9.233(8)	10.66(2)	10.79(2)	10.79(3)	10.77(2)
H_2L	17.462(9)	17.36(1)	17.323(9)	19.43(2)	20.53(1)	20.91(3)	21.28(2)
H_3L	-	-	-	27.44(2)	29.15(1)	29.74(2)	30.20(2)
H_4L	-	-	-	31.73(2)	36.90(1)	37.51(3)	37.99(1)
H_5L	-	-	-	-	41.41(2)	41.88(2)	42.45(2)
H_6L	-	-	-	-	45.31(2)	46.02(3)	46.20(3)
ZnH_2L	-	-	-	-	30.47(3)	30.44(7)	30.90(3)
ZnHL	-	-	-	20.30(2)	-	-	-
ZnL	10.10(3)	9.94(4)	9.98(2)	-	-	-	-
ZnH_4L_2	-	-	-	-	60.74(2)	60.46(6)	60.65(3)
ZnH_3L_2	-	-	-	-	50.85(4)	50.3(1)	50.79(9)
ZnH_2L_2	-	-	-	40.799(8)	40.82(3)	39.97(9)	40.43(9)
$ZnHL_2$	-	-	-	31.13(2)	-	-	29.5(2)
ZnL_2	19.86(2)	19.47(3)	19.17(2)	*n.d.*	*n.d.*	*n.d.*	*n.d.*

[a] $\beta M_iH_jL_k = [M_iH_jL_k]/([M]^i[H]^j[L]^k)$, where [L] is the concentration of fully deprotonated zinc hook peptide.

[b] Standard deviations are given as provided by SUPERQUAD calculations and refer to the last digit.

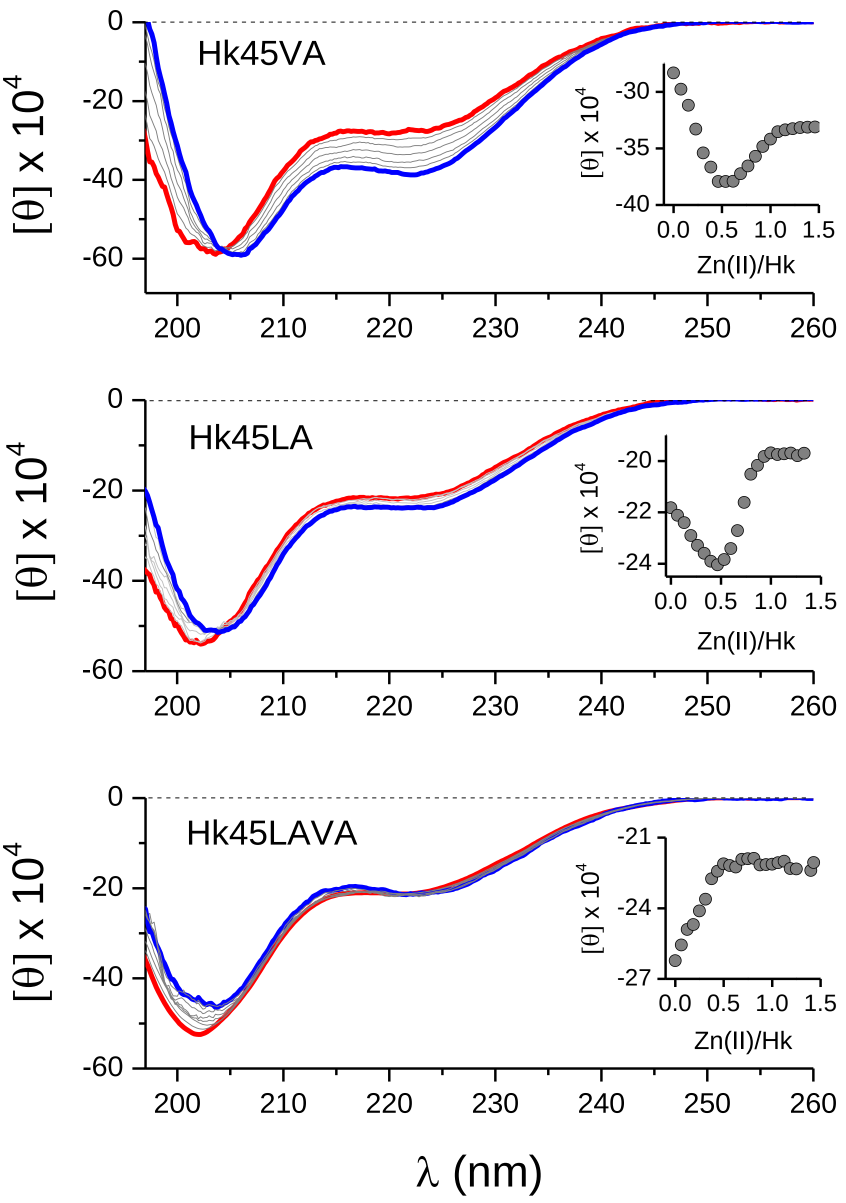

Figure S12. Spectropolarimetric titration of Hk45VA, Hk45LA, and Hk45LAVA zinc hook peptides with Zn^{2+} in 5 mM Tris buffer (I = 0.1 M from $NaClO_4$) at pH 7.4 and 25°C. Red and blue correspond to free peptide and a Zn^{2+}-to-peptide ratio of 0.5:1 (Zn(Hk)2 complex), respectively. Spectra collected at higher ratios are omitted for clarity. Insets present the dependence of the molar ellipticity of the zinc hook peptide on the Zn^{2+}-to-Hk peptide molar ratio. Ellipticity was monitored at either 202 nm (Hk45LAVA) or 222 nm (Hk45VA and Hk45LA).

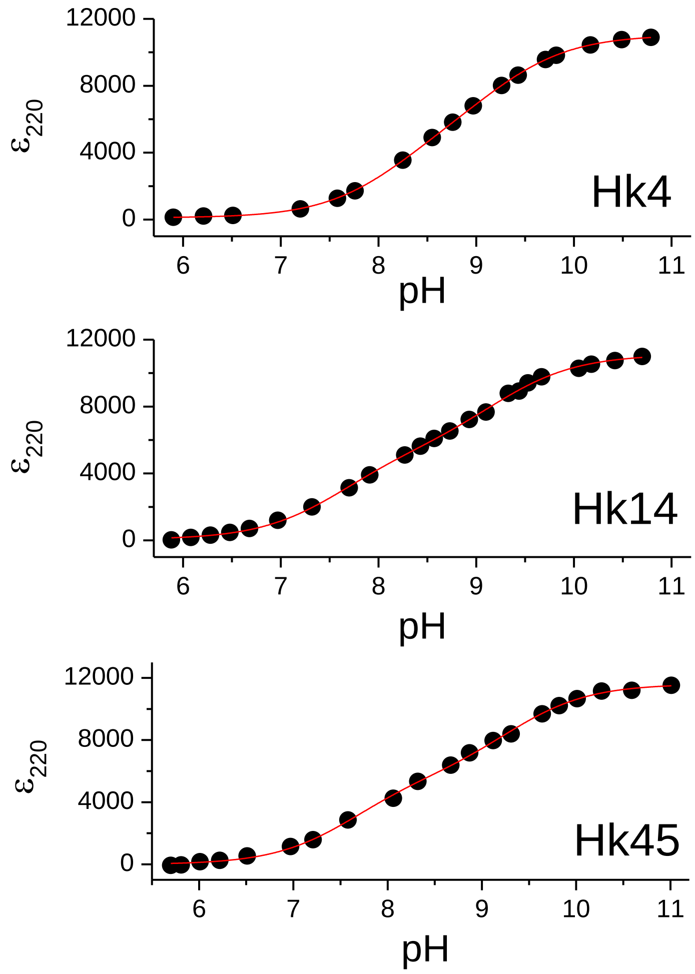

Figure S13. Examples of the pH titration of 100 µM metal-free zinc hook peptides in 0.1 M NaClO4, 25°C. Absorbance values were fitted to two-binding-event equations and represent the pK_{a1}^{SH} and pK_{a2}^{SH} values of cysteine residues.

Table S7. Dissociation constants of the cysteine thiols of zinc hook peptides determined potentiometrically and spectrophotometrically at I = 0.1 M and 25°C.[a] The pK_{a1}^{SH} and pK_{a1}^{SH} values allow the number of protons associated with Cys residues at pH 7.4 to be calculated.

Hook Peptide	Potentiometry		UV-vis		Number of protons at pH 7.4
	pK_{a1}^{SH}	pK_{a2}^{SH}	pK_{a1}^{SH}	pK_{a2}^{SH}	
Hk4	8.18 (2)	9.36(1)	8.19 (1)	9.28 (1)	1.86
Hk5	8.04 (2)	9.15 (2)	8.06 (2)	9.20 (2)	1.82
Hk6	7.81 (2)	8.98 (2)	7.92 (3)	9.18 (3)	1.76
Hk8	7.74 (5)	8.88 (4)	7.88 (2)	9.15 (2)	1.74
Hk10	7.36 (3)	8.89 (4)	7.60 (2)	9.19 (3)	1.60
Hk12	7.38 (6)	8.81 (5)	7.59 (2)	9.18 (4)	1.60
Hk14	7.39 (4)	8.99 (3)	7.56 (3)	9.17 (3)	1.58
Hk23	*n.a.*	*n.a.*	7.48 (3)	9.21 (3)	1.55
Hk27	*n.a.*	*n.a.*	7.50 (1)	9.16 (3)	1.55
Hk31	*n.a.*	*n.a.*	7.52 (1)	9.12 (4)	1.56
Hk37	*n.a.*	*n.a.*	7.55 (2)	9.14 (3)	1.57
Hk45	*n.a.*	*n.a.*	7.53 (2)	9.15 (2)	1.56

[a] Statistical errors of constant determinations are given in parentheses and refer to the last digit of the value.

Table S8. Average pK_a' values of the deprotonation of cysteine thiols of hook peptides in the presence of Zn^{2+} ions determined spectrophotometrically at 25°C and I = 0.1 M (see also Figure S14).

Mutated peptides	-logK_a' (pK_a') [a]
Hk4	6.25 ± 0.01
Hk5	6.16 ± 0.01
Hk6	5.79 ± 0.01
Hk8	5.53 ± 0.01
Hk10	5.24 ± 0.01
Hk12	5.15 ± 0.01
Hk14	5.08 ± 0.01
Hk23	[a]
Hk27	[a]
Hk31	[a]
Hk37	[a]
Hk45	4.70 ± 0.01

[a] Values not determined due to peptide precipitation at low pH.

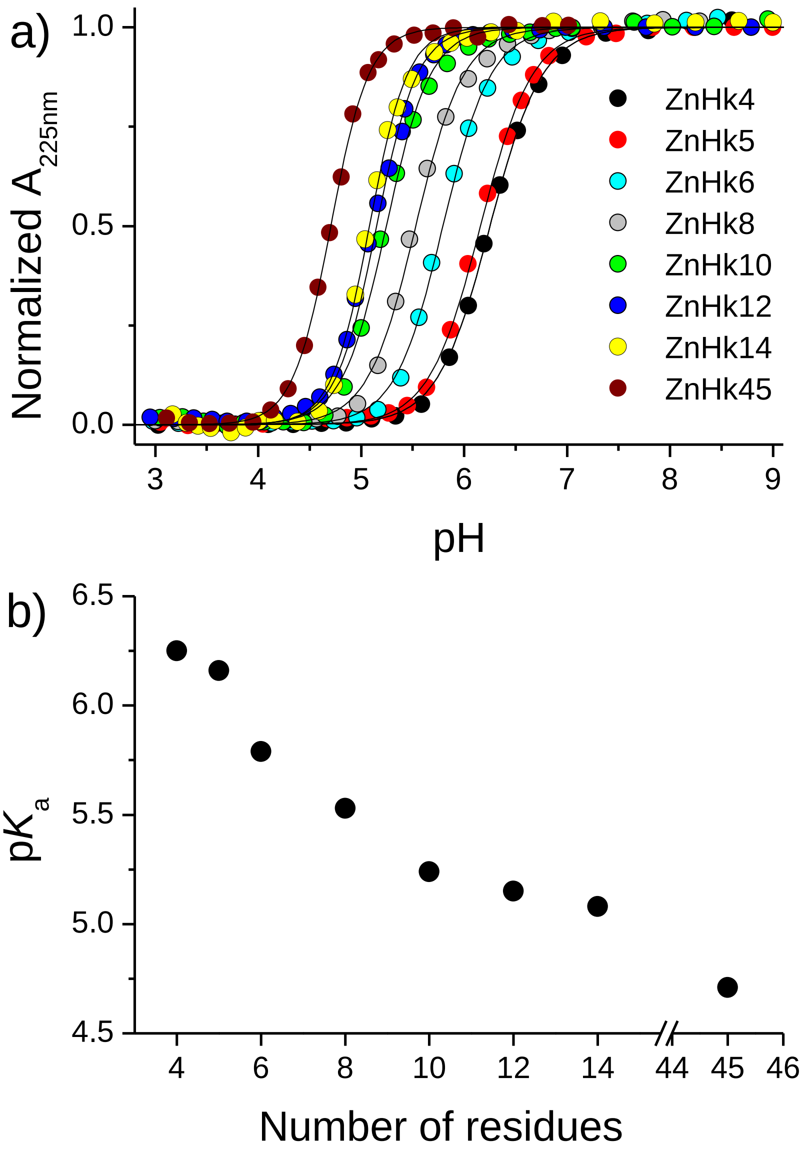

Figure S14. Determination of the apparent average thiol dissociation constants (pK_a') of zinc hook peptides in the presence of Zn^{2+} ions. a) Normalized absorbance at 220 nm corresponding to the formation of the LMCT bands of the complexes. b) Relationship between the length of the zinc hook peptide and pK_a' value.

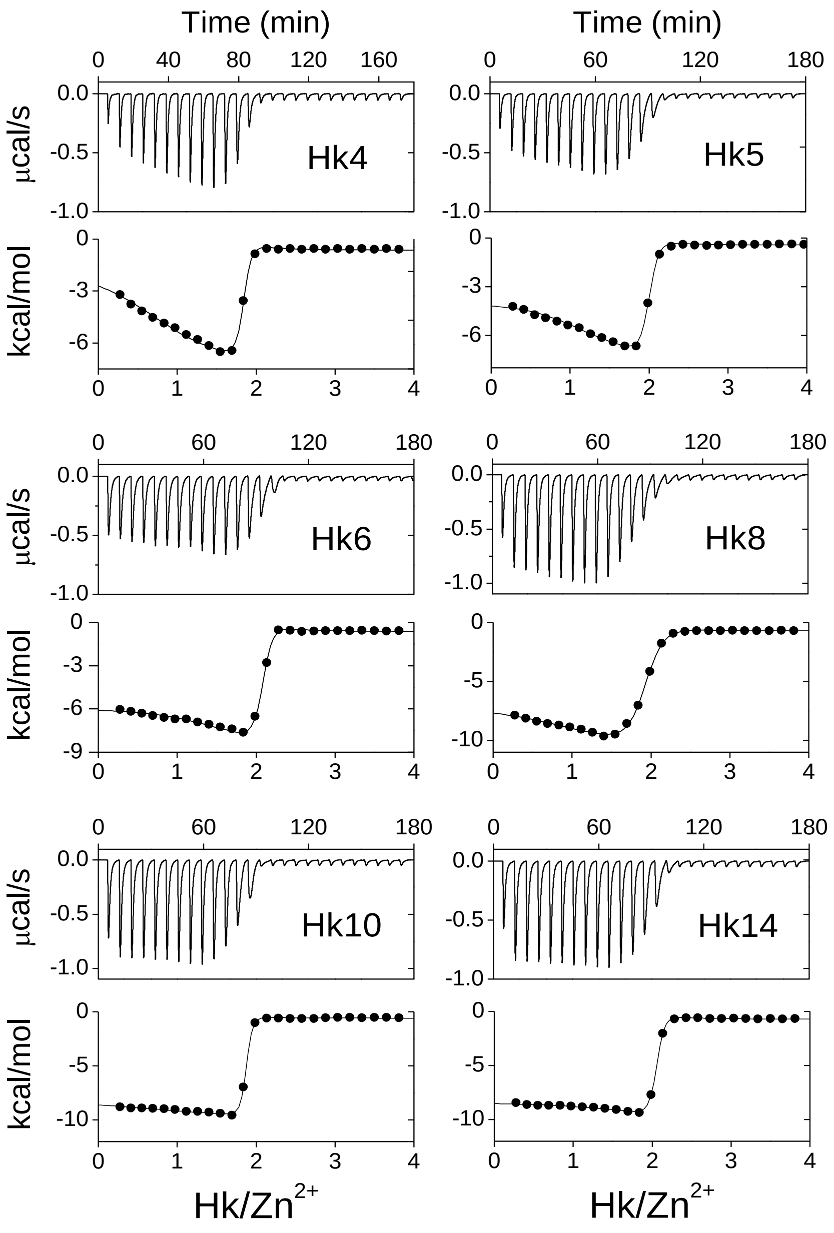

Figure S15. ITC traces of 50 µM Zn^{2+} titration with 1.3 mM zinc hook peptides in 50 mM HEPES, pH 7.4, 25°C. Data were fitted to the sequential binding model. The calculated enthalpies of $Zn(Hk)_2$ complex formation are presented in Table 1.

Table S9. Best fit values of ITC experiments. Traces for Hk4-Hk14 peptides were fitted to a binding model accounting for the formation of ZnHk and $Zn(Hk)_2$ complexes during the course of titration and Hk23-Hk45 were fitted to a binding model accounting for the formation of $Zn(Hk)_2$ complexes.

Peptide	Reaction 1: Zn^{2+} + Hk ⇆ ZnHk (K_1)		Reaction 2: Zn^{2+} + 2Hk ⇆ $Zn(Hk)_2$ (K_{12})	
	logK_1	$\Delta H_{ITC(1)}$ (kcal/mol)	logK_{12}	$\Delta H_{ITC(12)}$ (kcal/mol)
Hk4	7.0	-3.2	13.1	-12.1
Hk5	7.1	-3.8	13.5	-13.5
Hk6	6.9	-4.9	14.0	-15.2
Hk8	6.8	-7.9	13.0	-19.3
Hk10	6.8	-7.8	13.9	-18.2
Hk12	6.8	-7.8	14.0	-18.3
Hk14	6.0	-6.2	14.0	-18.2
Hk23	*nd*	*nd*	14.8	-17.7
Hk27	*nd*	*nd*	14.8	-18.1
Hk31	*nd*	*nd*	13.9	-19.5
Hk37	*nd*	*nd*	13.6	-20.6
Hk45	*nd*	*nd*	14.9	-22.5

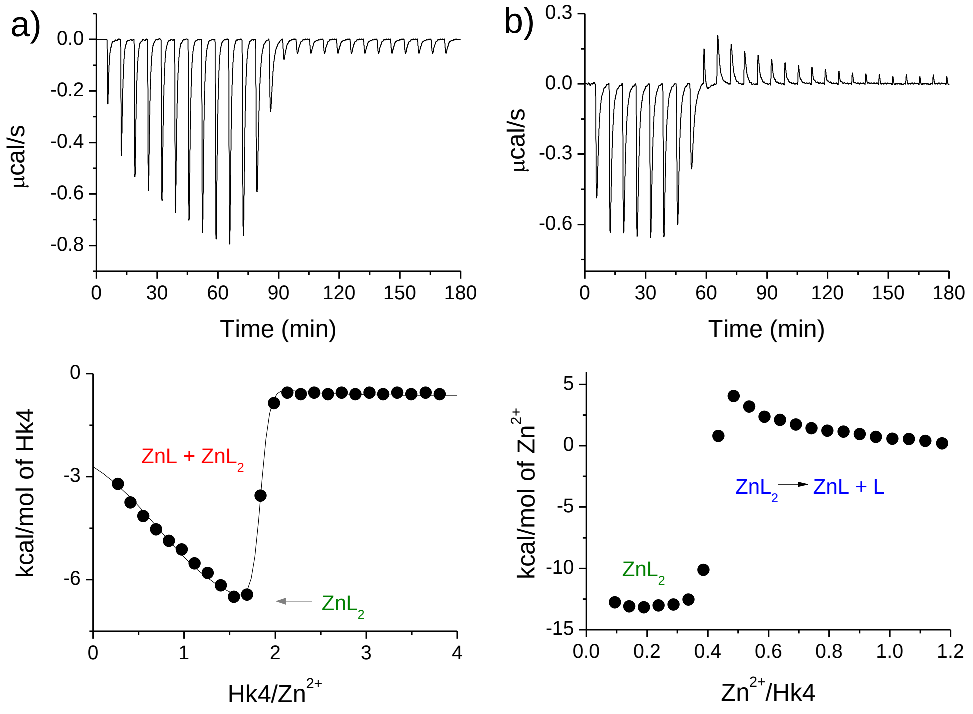

Figure S16. Comparison of ITC experiments of Zn-Hk4 system performed in both directions. A) Titration of Zn^{2+} with Hk4; b) titration of Hk4 with Zn^{2+}. Both experiments were performed in 50 mM HEPES buffer (I = 0.1 M), pH 7.4, 25°C. Red color shows titration range where both ZnL and ZnL_2 complexes are formed. Green color refers to the range where ZnL_2 complex is preferentially formed. Blue color indicates the range where ZnL_2 complex dissociates to ZnL upon addition of Zn^{2+} ions. Note that both titrations show full reversibility of ZnL and ZnL_2 complex formation.

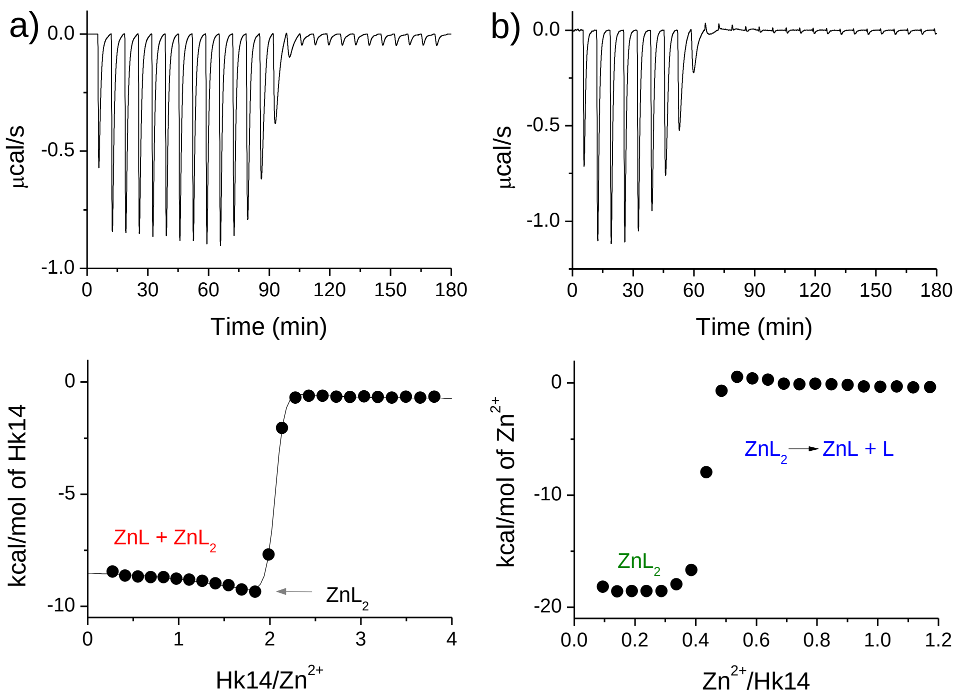

Figure S17. Comparison of ITC experiments of Zn-Hk14 system performed in both directions. A) Titration of Zn^{2+} with Hk14; b) titration of Hk14 with Zn^{2+}. Both experiments were performed in 50 mM HEPES buffer (I = 0.1 M), pH 7.4, 25°C. Red color shows titration range where both ZnL and ZnL_2 complexes are formed. Green color refers to the range where ZnL_2 complex is preferentially formed. Blue color indicates the range where ZnL_2 complex dissociates to ZnL upon addition of Zn^{2+} ions. Note that both titrations show full reversibility of ZnL and ZnL_2 complex formation.

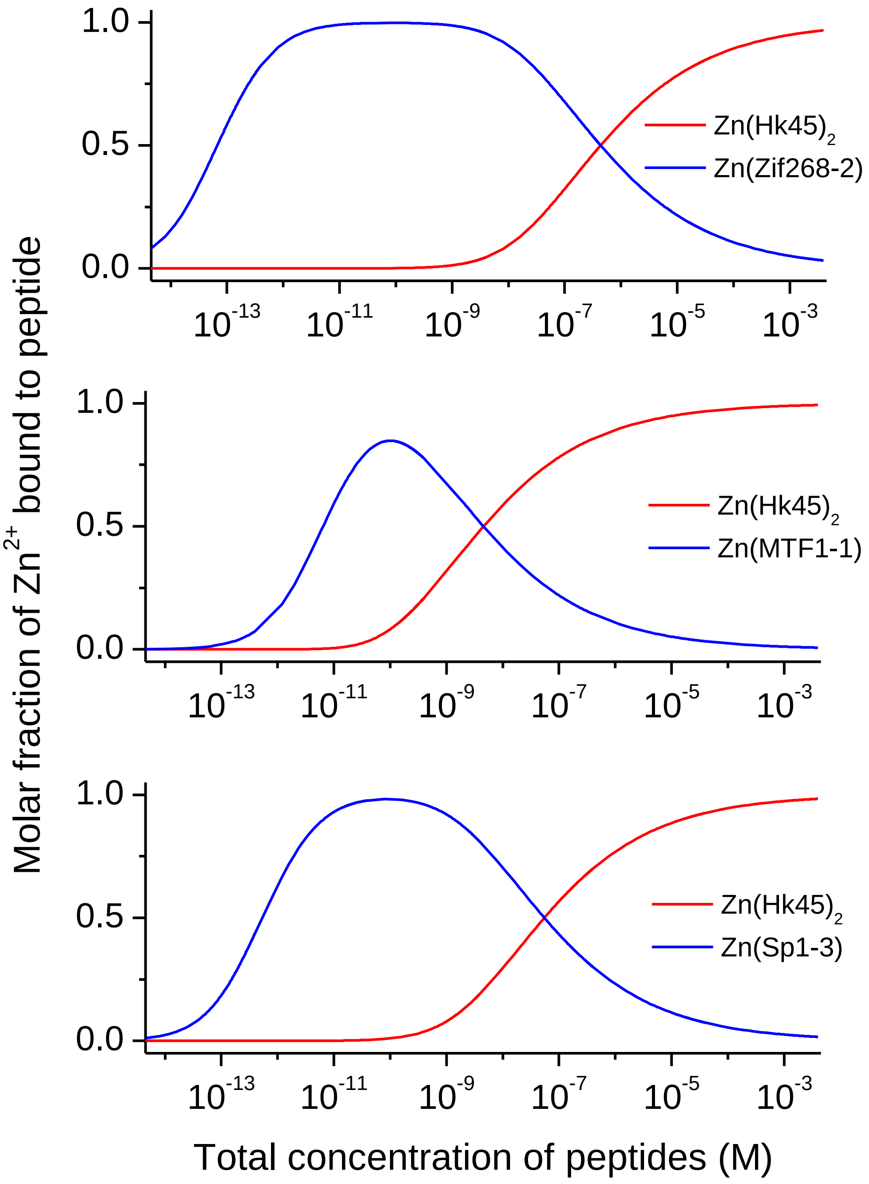

Figure S18. Speciation of Zn^{2+} complexes formed by equimolar amounts of zinc finger and zinc hook Hk45 peptides at various concentrations at pH 7.4.[22] a) Zif268-1 zinc finger (log$K$ = 13.6), Hk45 (logK_{12} = 20.74) and Zn^{2+} at molar ratio of 2:2:1; b) Sp1-3 zinc finger (log$K$ = 12.70), Hk45 and Zn^{2+} at molar ratio of 2:2:1. c) MTF1-1 zinc finger (log$K$ = 12.70), Hk45 and Zn^{2+} at molar ratio of 2:2:1.

Additional information for Experimental section

Table S10. The calculated (MW_{cal}) and experimentally determined (MW_{exp}) molecular masses of zinc hook peptides. Depending on the length of the peptide, the monoisotopic (m) or average (av) mass is listed.

Zinc hook peptide	Short name	MW_{cal}	MW_{exp}
Ac-CPVC-NH_2	Hk4	461.2^{m}	461.2^{m}
Ac-C**A**VC-NH_2	Hk4PA	435.2^{m}	435.3^{m}
Ac-CP**A**C-NH_2	Hk4VA	433.1^{m}	433.2^{m}
Ac-C**AA**C-NH_2	Hk4PAVA	407.1^{m}	407.2^{m}
Ac-CPVCG-NH_2	Hk5	518.2^{m}	518.5^{m}
Ac-CPVCGR-NH_2	Hk6	674.3^{m}	674.6^{m}
Ac-KCPVCGRE-NH_2	Hk8	931.4^{m}	931.7^{m}
Ac-KCPVC*GRE-NH_2	*depsi*Hk8	932.4^{m}	932.7^{m}
Ac-GKCPVCGREL-NH_2	Hk10	1102.4^{m}	1102.3^{m}
Ac-KGKCPVCGRELT-NH_2	Hk12	1331.6^{av}	1331.6^{av}
Ac-AKGKCPVCGRELTD-NH_2	Hk14	1517.8^{av}	1517.8^{av}
Ac-AKGKCPVC*GRELTD-NH_2	*depsi*Hk14	1518.8^{av}	1519.2^{av}
Ac-AKGKCPVC*GRE**A**TD-NH_2	*depsi*Hk14LA	1476.7	1476.6
Ac-AKGKCP**A**C*GRE**A**TD-NH_2	*depsi*Hk14VALA	1448.7	1448.4
Ac-LKKAKGKCPVCGRELTDEHREEL-NH_2	Hk23	2681.1^{av}	2679.2^{av}
Ac-EELKKAKGKCPVCGRELTDEHREEL-LS-NH2	Hk27	3140.3	3139.5^{av}
Ac-AIEELKKAKGKCPVCGRELTDEHREE-LLSKY-NH_2	Hk31	3615.1^{av}	3615.7^{av}
Ac-LKTAIEELKKAKGKCPVCGRELTDEH-REELLSKYHLD-NH_2	Hk37	4322.9^{av}	4323.4^{av}
Ac-KIGDLKTAIEELKKAKGKCPVCGRELT-DEHREELLSKYHLDLNNS-NH_2	Hk45	5164.8^{av}	5165.8^{av}
Ac-KIGDLKTAIEELKKAKGKCP**A**CGRELT-DEHREELLSKYHLDLNNS-NH_2	Hk45VA	5136.8^{av}	5136.7^{av}
Ac-KIGDLKTAIEELKKAKGKCPVCGRE**A**T-DEHREELLSKYHLDLNNS-NH_2	Hk45LA	5122.8^{av}	5123.3^{av}
Ac-KIGDLKTAIEELKKAKGKCP**A**CGRE**A**T-DEHREELLSKYHLDLNNS-NH_2	Hk45AA	5094.7^{av}	5095.4^{av}

Table S11. Relevant NMR experimental parameters.

Experiment (overall experiment time)	Dimension	Nucleus	Complex points of time domain	Acquisition time (ms)
$^{1}H/^{15}N$ HSQC (182 min)	t_1	^{15}N	128	58.2
	t_2	^{1}H	1024	85.2
$^{1}H/^{13}C$ HSQC (133 min)	t_1	^{13}C	128	6.1
	t_2	^{1}H	1024	85.2
TOCSY (116 min)	t_1	^{1}H	256	40.3
	t_2	^{1}H	541	85.3
ROESY (13 h 4 min)	t_1	^{1}H	256	40.3
	t_2	^{1}H	541	85.3

References:

1 J. Eildal, G. Hultqvist, T. Balle, N. Stuhr-Hansen, S. Padrah, S. Gianni, K. Strømgaard and P. Jemth, *J. Am. Chem. Soc.*, 2013, **135**, 12998-13007.
2 P. Eyer, F. Worek, D. Kiderlen, G. Sinko, A. Stuglin, V. Simeon-Rudolf and E. Reiner, *Anal. Biochem.*, 2003, **312**, 224-227.
3 A. Krężel, R. Latajka, G. Bujacz and W. Bal, *Inorg. Chem.*, 2003, **42**, 1994-2003.
4 P. Gans, A. Sabatini and A. Vacca, *J. Chem. Soc., Dalton Trans.*, 1985, **6**, 1195.
5 A. Krężel and W. Maret, *Arch. Biochem. Biophys.*, 2016, doi: 10.1016/j.abb.2016.04.010.
6 L. Alderighi, P. Gans, A. Ienco, D. Peters, A. Sabatini and A. Vacca, *Coord. Chem. Rev.*, 1999, **184**, 311-318.
7 M. Sikorska, A. Krężel and J. Otlewski, *J. Inorg. Biochem.*, 2012, **115**, 28-35.
8 J. Adamczyk, W. Bal and A. Krężel, *Inorg. Chem.*, 2015, **54**, 596-606.
9 A. Krężel and W. Bal, *J. Inorg. Biochem.*, 2004, **98**, 161-166.
10 S. Forsén and R. Hoffman, *J. Chem. Phys.*, 1963, **39**, 2892.
11 J. Wójcik, K. Ruszczyńska, I. Zhukov and A. Ejchart, *Acta Biochim. Polon.*, 1998, **46**, 651-663.
12 Y. Bai, J. Milne, L. Mayne and S. Englander, *Proteins Structure Funct. Bioinf.* 1993, **17**, 75-86.
13 L. Braunschweiler and R. Ernst, *J. Magn. Reson.*, (1969), 1983, **53**, 521-528.
14 A. Bax and D. Davis, *J. Magn. Reson.*, (1969), 1985, **63**, 207-213.
15 G. Bodenhausen and D. Ruben, *Chem. Phys. Lett.*, 1980, **69**, 185-189.
16 D. Marion, M. Ikura, R. Tschudin and A. Bax, *J. Magn. Reson.*, (1969), 1989, **85**, 393-399.
17 D. Wishart, C. Bigam, J. Yao, F. Abildgaard, H. Dyson, E. Oldfield, J. Markley and B. Sykes, *J. Biomol. NMR*, 1995, **6**. 135-140.
18 T. Goddard and D. Kneller, SPARKY 3, *University of California, San Francisco* 15, 2004
19 T. Herrmann, P. Güntert and K. Wüthrich, *J. Biomol. NMR*, 2002, **24**, 171-189.
20 K. Hopfner, L. Craig, G. Moncalian, R. Zinkel, T. Usui, B. Owen, A. Karcher, B. Henderson, J. Bodmer, C. McMurray, J. Carney, J. Petrini and J. Tainer, *Nature*, 2002, **418**, 562-566.
21 G. Vriend, *J. Mol. Graph.*, 1990, **8**, 52-56.
22 T. Kochańczyk, A. Drozd and A. Krężel, *Metallomics*, 2015, **7**, 244-257.
